# Ectopic Pregnancy as a Model to Identify Endometrial Genes and Signaling Pathways Important in Decidualization and Regulated by Local Trophoblast

**DOI:** 10.1371/journal.pone.0023595

**Published:** 2011-08-17

**Authors:** W. Colin Duncan, Julie L. V. Shaw, Stewart Burgess, Sarah E. McDonald, Hilary O. D. Critchley, Andrew W. Horne

**Affiliations:** 1 MRC Centre for Reproductive Health, The University of Edinburgh, Edinburgh, Midlothian, United Kingdom; 2 Moredun Research Institute, Penicuik, Midlothian, United Kingdom; Kyushu Institute of Technology, Japan

## Abstract

The endometrium in early pregnancy undergoes decidualization and functional changes induced by local trophoblast, which are not fully understood. We hypothesized that endometrium from tubal ectopic pregnancy (EP) could be interrogated to identify novel genes and pathways involved in these processes. Gestation-matched endometrium was collected from women with EP (n = 11) and intrauterine pregnancies (IUP) (n = 13). RNA was extracted from the tissue. In addition, tissues were prepared for histological analysis for degree of decidualization. We compared a) the samples from EP that were decidualized (n = 6) with non-decidualized samples (n = 5), and b) the decidualized EP (n = 6) with decidualization-matched IUP (n = 6) samples using an Affymetrix gene array platform, with Ingenuity Pathway Analysis, combined with quantitative RT-PCR. Expression of *PRL* and *IGFBP1* was used to confirm the degree of decidualization in each group. There were no differences in *PRL* or *IGFBP1* expression in the decidualization-matched samples but a marked reduction (*P*<0.001) in the non-decidualized samples. Decidualization was associated with increased expression of 428 genes including *SCARA5* (181-fold), *DKK1* (71-fold) and *PROK1* (32-fold), and decreased expression of 230 genes including *MMP-7* (35-fold) and *SFRP4* (21-fold). The top canonical pathways associated with these differentially expressed genes were Natural Killer Cell and Wnt/b-Catenin signaling. Local trophoblast was associated with much less alteration of endometrial gene expression with an increase in 56 genes, including *CSH1* (8-fold), and a reduction in 29 genes including *CRISP3* (8-fold). The top associated canonical pathway was Antigen Presentation. The study of endometrium from tubal EP may promote novel insights into genes involved in decidualization and those influenced by factors from neighboring trophoblast. This has afforded unique information not highlighted by previous studies and adds to our understanding of the endometrium in early pregnancy.

## Introduction

Transformation of the endometrial lining of the uterus into decidua is an essential requirement for blastocyst implantation in women [1]. Normal placentation will fail in the absence of endometrial decidualization [2]. Although decidualization is of fundamental importance in human fertility its regulation is still not fully understood at a molecular level. In women, unlike other mammalian species, the decidual transformation of the endometrium does not require blastocyst attachment and invasion. It commences in the secretory phase of the menstrual cycle under the influence of progesterone and continues if pregnancy occurs [3]. However it is still not clear what role local trophoblast factors may have in this process.

We hypothesized that the presence of trophoblast alters decidual function and that gene expression in the decidualized endometrium would therefore be different in an ectopic pregnancy (EP) compared to an intrauterine pregnancy (IUP) and this difference could be used to help diagnose EP. We therefore investigated decidual gene expression, and secretory function, in a biomarker discovery program [4], [5]. We have discovered that activin B is a decidual product that is lower in tubal ectopic pregnancy [4]. However, in that study we found that the overall degree of decidualization was variable in tubal EP and overall less than that seen in IUP [4]. In addition, we discovered that cystein-rich secretory protein 3 (*CRISP3*) expression was increased in the decidualized endometrium of tubal ectopic pregnancy when compared to intrauterine pregnancies [5]. It was reported that, rather than being related to decidualization, CRISP3 was related to human chorionic gonadotropin (hCG) concentrations and that hCG could inhibit endometrial cell CRISP3 expression *in vitro* [5]. The endometrial changes in early pregnancy therefore involve decidualization and changes secondary to the paracrine effects of local trophoblast.

Dissecting the relative changes secondary to decidualization and trophoblast invasion is challenging. Decidualization of the endometrium does occur in ectopic pregnancy [4], [6] and similarities in the cellular composition [7] and leukocyte subtypes [8] of uterine decidua in tubal and intrauterine pregnancies have been described. We therefore hypothesized that tubal ectopic pregnancy provides a useful model for the examination of the decidualized endometrium of pregnancy where there is no local trophoblast influence. We further hypothesized that tubal ectopic pregnancy could be used to investigate the paracrine effect of local trophoblast on the decidua to further our understanding of the genes involved in decidualization, embryo implantation and the establishment of early pregnancy.

Herein, we report the use of microarray technology and pathway analysis on the endometrium of tubal ectopic pregnancies with different degrees of decidual change as a paradigm to identify novel genes involved in decidualization in early pregnancy. In addition, using endometrium from women with ectopic and intrauterine pregnancies, with equivalent decidualization, we have applied the same technology to discover genes regulated by local trophoblast.

## Results

### Microarray analysis of the decidualized endometrium from intrauterine and tubal ectopic pregnancies

**Figure 1:**
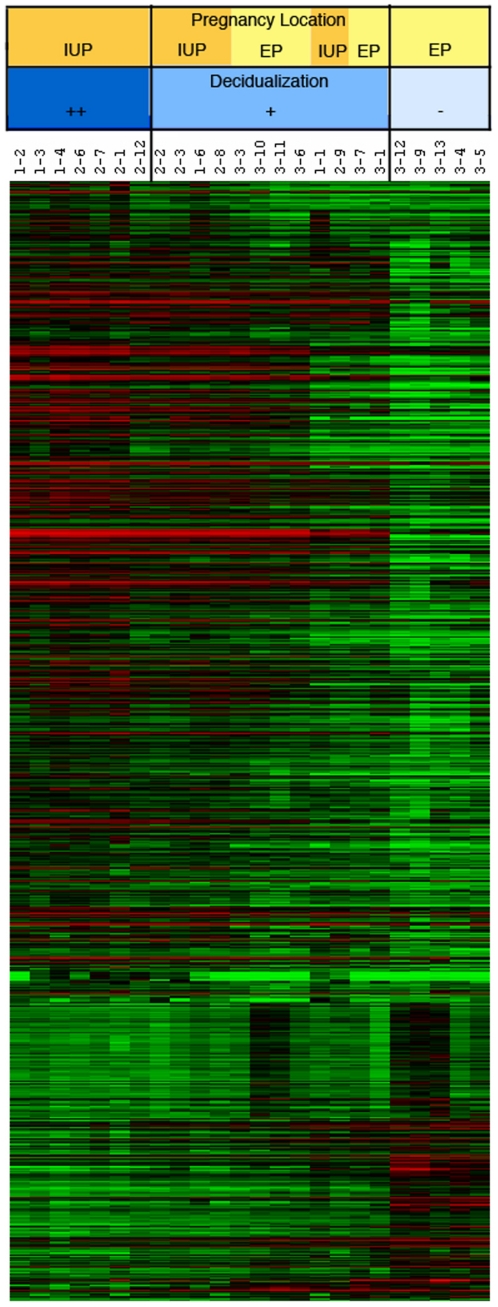
Heat map showing hierarchical clustering of individual arrays by gene expression. Hierachical clustering was performed on the decidua from women with ectopic pregnancy (EP) and intrauterine pregnancy (IUP) on entities (genes) using the Pearson Centroid method in Array Assist. The genes used in this comparison (∼14,000) had passed an initial filtering stage to filter out genes with very low absolute levels of expression (<64) in 50% of the arrays. Samples are clustered into 3 groups based on degree of decidualization. The most decidualized (++) IUP cluster together as do the non-decidualized (−) EP. Between these is a mixture of partially decidualized (+) IUP and EP samples. The numbers are the codes for the samples (IUP; 1- is from viable intrauterine [termination of pregnancy] and 2- is from non-viable intrauterine pregnancy [miscarriage]. EP; 3- is from tubal ectopic pregnancy).

Protocols of the experimental procedures, methods of analysis and microarray data are available as supplementary information in the European Bioinformatics Institute's MIAME compliant ArrayExpress database http://www.ebi.ac.uk/arrayexpress website: accession number E-MTAB-680. Heat maps based on average fold-changes (FC) for each gene in the array were generated to visualize the level of correlation between the individual samples and sample groups. The arrays demonstrated a good degree of correlation between samples with values between 0.85–0.99. Hierarchical clustering analysis revealed that samples clustered based on their degree of decidualization (Fig. 1). It was clear that one cluster represented the highly decidualized samples and these were all from intrauterine pregnancies. Another cluster represented the samples with little or no decidualization and these were all from ectopic pregnancies. The other samples displayed moderate decidualization and represented a mixture of intrauterine and ectopic pregnancies.

**Figure 2:**
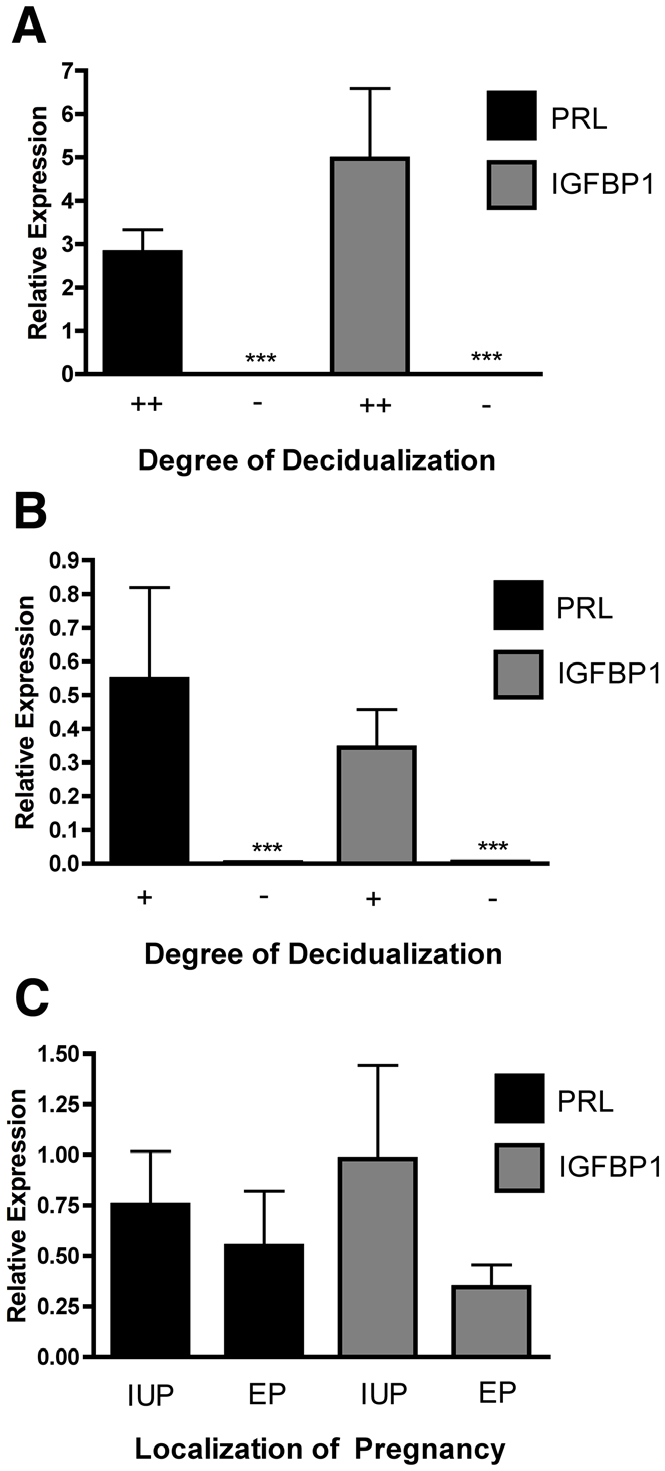
Expression of markers of decidualization. PRL and IGFBP1 are known markers of endometrial decidualization. A) Both *PRL* (*P*<0.001) and *IGFBP1* (*P*<0.001) were higher in the decidualized samples (++) (n = 7) than the non-decidualized samples (−) (n = 5). B) In tubal ectopic pregnancy (EP) both *PRL* (*P*<0.001) and *IGFBP1* (*P*<0.001) were higher in the partially decidualized samples (+) (n = 6) than the non-decidualized samples (−) (n = 5). C) When standardized for decidualization (+) there was no difference in the expression of *PRL* or *IGFBP1* in samples from EP (n = 6) and intrauterine pregnancy (IUP) (n = 6).

In order to validate the assessment of the decidualization status of the samples we performed qRT-PCR for *PRL* and *IGFBP1*, genes known to be markedly up-regulated during decidualization (Fig. 2). The expression of these genes was much higher in the highly decidualized biopsies than the samples showing little or no decidualization (Fig. 2A) (*P*<0.001). In order to investigate genes involved in decidualization we aimed to compare the samples from ectopic pregnancies with moderate decidualization with those with little or no decidualization (Fig. 2B). In ectopic pregnancies, the more decidualized samples also had higher expression of *PRL* and *IGFBP1* (Fig. 2B) (*P*<0.001). In order to assess the local effects of trophoblast we aimed to compare endometrial biopsies from intrauterine pregnancies with those from ectopic pregnancies with the same degree of decidualization (Fig. 2C). There was no difference in the expression of these genes in the moderately decidualized samples from intrauterine and ectopic pregnancies (Fig. 2C).

### Utilizing decidua from women with ectopic pregnancy to discover novel decidualization-associated genes

We analyzed the array results from the decidua from women with ectopic pregnancies with little or no decidualization and compared them to decidua from women with ectopic pregnancies with moderate decidualization (Table 1). We found that 658 genes were differentially expressed (FC≥2, *P*<0.05) depending on the degree of endometrial decidualization. All genes that showed a FC of ≥10-fold up (28 out of 428 up-regulated genes associated with decidualization) or down (15 out of 230 down-regulated genes) are shown in Table 1 and Table S1 lists all 658 genes with a FC of ≥2. Ingenuity pathway analysis (www.ingenuity.com) of these genes with a *P*-value cut-off of <0.001 found 36 significant pathways. The top canonical pathways associated with these genes are natural killer cell signaling and Wnt/ß-catenin signaling (Table 1) and the top 20 pathways are listed in Table S2.

**Table 1:** Endometrial genes and pathways associated with decidualization.

Gene Name	Gene	Fold Change
*Up-regulated genes*		
scavenger receptor class A, member 5	SCARA5	+181.0
dickkopf homolog 1 (Xenopus laevis)	DKK1	+70.71
prolactin	PRL	+47.06
chordin-like 1	CHRDL1	+31.62
prokineticin 1	PROK1	+18.94
ankyrin repeat domain 55	ANKRD55	+17.51
TIMP metallopeptidase inhibitor 3	TIMP3	+17.02
ST6 (alpha-N-acetyl-neuraminyl-2,3-beta-galactosyl-1,3)-N-acetylgalactosaminide alpha-2,6-sialyltransferase 5	ST6GALNAC5	+15.46
Collectin sub-family member 11	COLEC11	+14.46
complement factor D (adipsin)	CFD	+14.15
gastrin	GAST	+14.12
apolipoprotein D	APOD	+13.98
cytochrome P450, family 2, subfamily C, polypeptide 9	CYP2C9	+13.95
solute carrier family 18 (vesicular monoamine), member 2	SLC18A2	+13.74
aldehyde oxidase 1	AOX1	+13.58
fibroblast growth factor 7 (keratinocyte growth factor)	FGF7	+13.46
neuron navigator 3	NAV3	+13.17
cytochrome P450, family 4, subfamily B, polypeptide 1	CYP4B1	+12.79
aldehyde dehydrogenase 8 family, member A1	ALDH8A1	+12.76
angiopoietin-like 1	ANGPTL1	+12.68
cannabinoid receptor 1 (brain)	CNR1	+12.05
glypican 3	GPC3	+11.91
ADAM metallopeptidase with thrombospondin type 1 motif, 5	ADAMTS5	+11.47
family with sequence similarity 5, member B	FAM5B	+11.45
metallothionein 1 M	MT1M	+11.37
solute carrier family 18 (vesicular monoamine), member 2	SLC18A2	+11.36
arachidonate 12-lipoxygenase	ALOX12	+11.27
gamma-aminobutyric acid (GABA) A receptor, alpha 2	GABRA2	+11.07
UDP-N-acetyl-alpha-D-galactosamine:polypeptide N-acetylgalactosaminyltransferase 13 (GalNAc-T13)	GALNT13	+10.80
killer cell immunoglobulin-like receptor, two domains, short cytoplasmic tail, 1	KIR2DS1	+10.64
*Down-regulated genes*		
matrix metallopeptidase 7	MMP7	−35.26
secreted frizzled-related protein 4	SFRP4	−21.09
solute carrier family 47, member 1	SLC47A1	−20.28
deiodinase, iodothyronine, type II	DIO2	−17.77
matrix metallopeptidase 11 (stromelysin 3)	MMP11	−16.54
chemokine (C-X-C motif) ligand 2	CXCL2	−14.97
inhibin, beta A	INHBA	−13.13
serpin peptidase inhibitor, clade A (alpha-1 antiproteinase, antitrypsin), member 1	SERPINA1	−13.03
thymosin beta 15a	TMSB15A	−12.71
microfibrillar-associated protein 2	MFAP2	−12.56
secreted frizzled-related protein 1	SFRP1	−11.62
matrix-remodelling associated 5	MXRA5	−11.21
leucine rich repeat containing 17	LRRC17	−10.84
prostate transmembrane protein, androgen induced 1	PMEPA1	−10.72
contactin 1	CNTN1	−10.22

Canonical Pathway	*P* value	Ratio
Natural killer cell signaling	2.97 E-13	25/112 (0.223)
Wnt/b-catenin signaling	9.11 E-06	20/169 (0.118)
Crosstalk between dendritic cells and natural killer cells	1.54 E-05	14/98 (0.143)
Melatonin signaling	8.10 E-05	11/77 (0.143)
Role of macrophages, fibroblasts and endothelial cells in rheumatoid arthritis	1.14 E-04	28/357 (0.078)

The top genes and pathways identified in genomic arrays of partially decidualized endometrium compared to non-decidualized endometrium from ectopic pregnancy. A positive fold change shows that the genes are increased in the more decidualized samples and suggests the gene is increased in decidualization. A negative fold change shows that the genes are decreased in the more decidualized samples and suggests the gene is decreased in decidualization.

**Figure 3:**
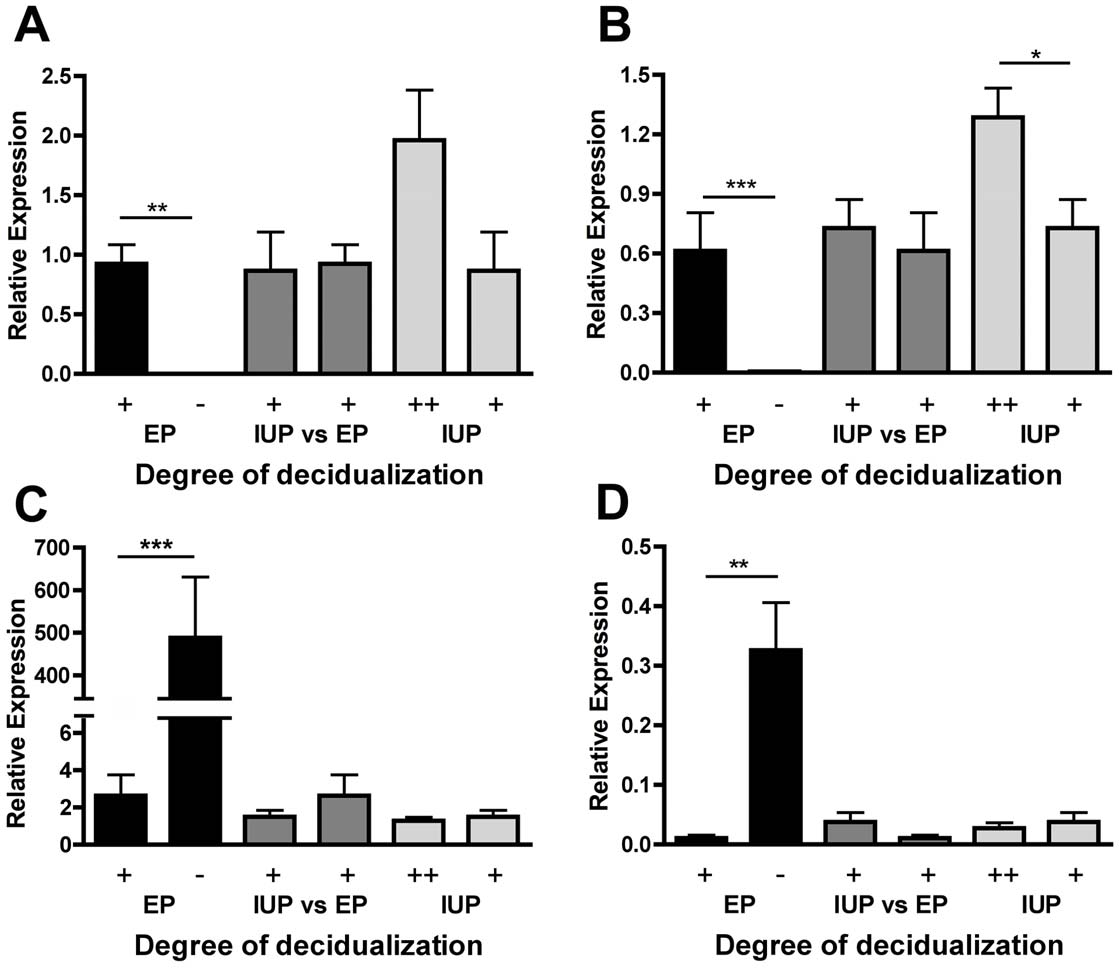
Expression of decidualization-related genes. Expression of genes in partially decidualized (+) (n = 6) and non-decidualized (−) (n = 5) endometrium from ectopic pregnancy (EP) and in the partially decidualized (+) (n = 6) and decidualized (++) (n = 7) endometrium from intrauterine pregnancy (IUP). A) *SCARA5* was higher in partially decidualized EP than non-decidualized EP samples (*P*<0.01). B) *DKK1* was higher in partially decidualized EP than non-decidualized EP samples (*P*<0.001) and in decidualized IUP than in partially decidualized IUP (*P*<0.05). In contrast, C) *MMP7* (*P*<0.001) and D) *INHBA* (*P*<0.01) were increased in the non-decidualized EP samples when compared to the partially decidualized EP endometrium.

Unsurprisingly *PRL* was increased in the more decidualized samples. The greatest up-regulation in expression associated with decidualization was seen for *SCARA5* (Fig. 3A) (*P*<0.01) and *DKK1* (Fig. 3B) (*P*<0.001). Neither of these genes showed differential expression between intrauterine or ectopic pregnancies of similar decidualization (Fig. 3A,B). Expression of these two genes was generally higher in the highly decidualized intrauterine endometrium when compared to the moderately decidualized endometrium (Fig. 3A,B). Other notable markedly up-regulated genes in the more decidualized endometrium include *PROK1*, *TIMP3*, *FGF7* and *ADAMTS5*.

Of the genes whose expression was reduced in the more decidualized samples (Table 1) the largest down-regulation was seen for *MMP7* (Fig. 3C) (*P*<0.001). There was no difference in *MMP7* expression between ectopic and intrauterine endometrium of similar decidualization (Fig. 3C). Other notable markedly down-regulated genes in the more decidualized endometrium include *INHBA* (Fig. 3D) (*P*<0.01), *SFRP4*, *SFRP1* and *MMP11*.

### Utilizing decidua from women with ectopic pregnancy to discover novel trophoblast-regulated endometrial genes

We analyzed the array results from decidua from women with ectopic pregnancies with moderate decidualization and compared them to those from women with intrauterine pregnancies with the same degree of decidualization (Table 2). We found that 85 genes were differentially expressed (FC≥2, *P*<0.05) depending on the pregnancy localization. All genes that showed a FC of ≥4-fold up (8 out of 56 genes up-regulated where there is local trophoblast) or down (4 out of 29 down-regulated genes) are shown in Table 2 and Table S3 lists all 85 genes with a FC≥2. Ingenuity pathway analysis of these genes with a *P*-value cut-off of <0.001 found 5 significant pathways (Table 2). The top canonical pathway associated with these genes is the antigen presentation pathway (Table 2).

**Table 2:** Endometrial genes and pathways associated with local trophoblast.

Gene Name	Gene	Fold Change
*Up-regulated genes*		
chorionic somatomammotropin hormone 1 (placental lactogen)	CSH1	+7.63
chorionic somatomammotropin hormone 2	CSH2	+7.44
carboxypeptidase A3 (mast cell)	CPA3	+5.66
glycoprotein hormones, alpha polypeptide	CGA	+5.44
chorionic somatomammotropin hormone-like 1	CSHL1	+4.97
forkhead box Q1	FOXQ1	+4.94
tryptase alpha/beta 1	TPSAB1	+4.86
tryptase beta 2	TPSB2	+4.55
*Down-regulated genes*		
cysteine-rich secretory protein 3	CRISP3	−8.16
phosphoserine phosphatase	PSPH	−6.10
uroplakin 1B	UPK1B	−4.40
gastrin	GAST	−4.22

Canonical Pathway	*P* value	Ratio
Antigen presentation pathway	2.35 E-03	4/39 (0.103)
Hepatic fibrosis/hepatic stellate cell activation	4.07 E-03	7/134 (0.052)
VDR/RXR activation	7.21 E-03	5/80 (0.062)
Pathogenesis of multiple sclerosis	8.21 E-03	2/9 (0.222)
ATM signaling	8.30 E-03	4/53 (0.075)

The top genes and pathways identified in genomic arrays of partially decidualized endometrium from intrauterine pregnancy compared to decidualization-matched endometrium from ectopic pregnancy. A positive fold change shows that the genes are increased in the samples from IUP and suggests the gene is increased where there is local trophoblast. A negative fold change shows that the genes are decreased in the samples from IUP and suggests the gene is decreased with the presence of local trophoblast.

**Figure 4:**
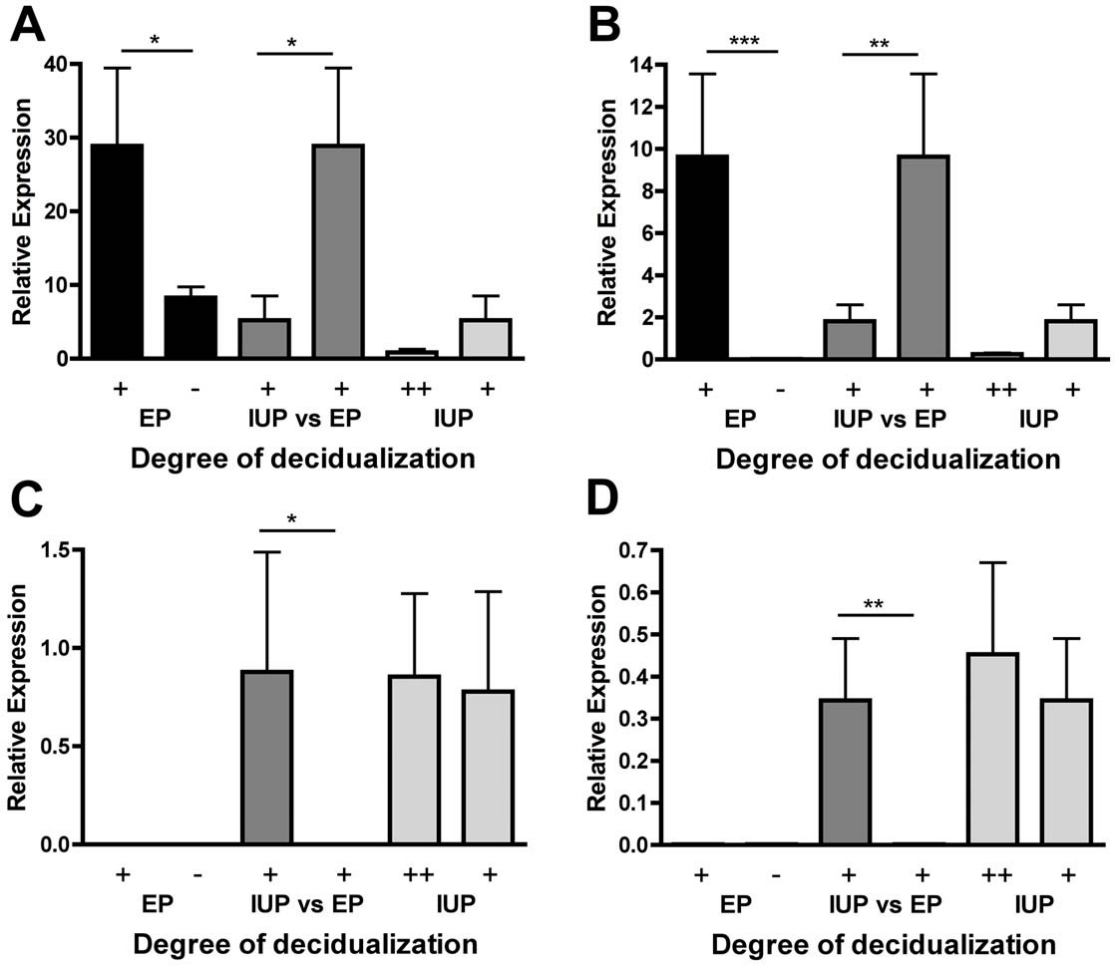
Expression of trophoblast-regulated endometrial genes. Expression of genes in partially decidualized (+) (n = 6) and non-decidualized (−) (n = 5) endometrium from ectopic pregnancy (EP) and in the partially decidualized (+) (n = 6) and decidualized (++) (n = 7) endometrium from intrauterine pregnancy (IUP). A) *CRISP3* was lower (*P*<0.05) in endometrium from IUP when compared to that from decidualization matched EP as is B) *GAST* (P<0.01). However both A) *CRISP3* (*P*<0.05) and B) *GAST* (*P*<0.001) were increased by partial decidualization in EP. In contrast, C) *CSH1* (*P*<0.05) and D) *CGA* (*P*<0.01) were increased in the partially-decidualized IUP samples when compared to the partially decidualized EP endometrium.

Of the genes whose expression was reduced by the presence of local trophoblast the largest down-regulation was seen for *CRISP3* (Fig. 4A) (*P*<0.05). As well as being more highly expressed in decidualization-matched endometrium from ectopic pregnancies there also seemed to be an effect of decidualization on *CRISP3* expression (Fig. 4A) (*P*<0.05). In decidua from women with ectopic pregnancy, decidualization increased *CRISP3* expression. Another notable gene showing a similar pattern was *GAST* (Fig. 4B). *GAST* expression was reduced by the presence of local trophoblast (*P*<0.01). In addition *GAST* expression was also increased by decidualization (Fig. 4B) (*P*<0.001) as expected from Table 1.

**Figure 5:**
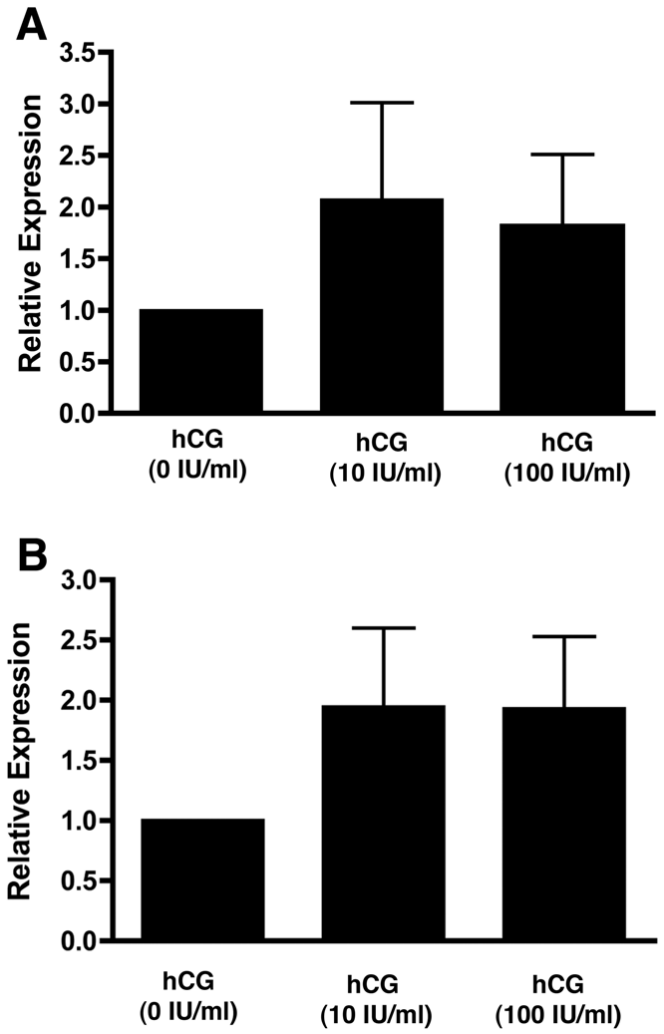
The effect of hCG on trophoblast-regulated genes in an endometrial epithelial cell line. Expression of both A) *CSH1* and B) *CGA* could be detected in hTERT-EEpC cells but the trend to increase after treatment with 10 IU/ml or 100 IU/ml did not reach statistical significance.

Of the genes whose expression was increased by the presence of local trophoblast *CSH1* (Fig. 4C) (*P*<0.05) and *CSH2* showed the largest increase. Other interesting genes that showed increased expression included *CGA* (Fig. 4D) (*P*<0.01) and the enzymes *CPA3*, *TPSAB1* and *TPSB2*. It is notable that many of these genes are trophoblast-associated. This raised the possibility of microscopic trophoblast contamination that was not seen in the immunohistochemical analysis. We therefore investigated the effect of hCG on endometrial epithelial cell expression of *CSH1* and *CGA in vitro* (Fig. 5). Endometrial epithelial cells did express *CSH1* (Fig. 5A) and *CGA* (Fig. 5B) and although hCG tended to increase expression this did not reach statistical significance.

## Discussion

Endometrial decidualization has a fundamental role in the establishment of human pregnancy [1], [2]. Increasing our understanding of the genes and pathways involved in this key process will facilitate the development of strategies to manipulate endometrial function in order to promote normality or inhibit fertility. While there are established *in vitro* models of decidualization [4], [9] these lack the cellular complexity and paracrine interactions that occur *in vivo*. *In vivo* assessment of decidual changes in early pregnancy is challenging [10], [11] as there are difficulties in controlling for temporal and local factors. In addition, it is not known if trophoblast locally affects or regulates the process of decidualization in early pregnancy or indeed what paracrine effects the trophoblast may have on the endometrium.

We have previously established that decidualization of the endometrium in tubal ectopic pregnancies is variable [4]. In this study, we collected gestation-matched endometrium from ectopic pregnancy and stratified for degree of decidualization to assess genes involved in decidualization in early pregnancy in the absence of the local effects of trophoblast. In addition, using the same strategy, using gestation matched ectopic and intrauterine pregnancies stratified for degree of decidualization, we assessed the contribution of local trophoblast to endometrial function. We used microarray analysis to interrogate these samples and to highlight novel endometrial genes associated with decidualization and those regulated by trophoblast.

The use of microarray technology to assess endometrial decidualization is not novel. Popovici *et al.* used microarrays to examine genes differentially expressed in human endometrial stromal cells decidualized *in vitro* [9]. They reported changes in numerous genes including an up-regulation of cytokines, chemokines and growth factors [9]. Chen *et al.* examined genes differentially expressed between the decidua and trophoblast of early human pregnancy [12]. They highlighted differences in extracellular matrix proteins and cell adhesion molecules [12]. Other studies have focussed on the effects of exogenous molecules on cultured human decidual cells [13], [14]. Although decidua from ectopic pregnancy has been compared against mid-secretory endometrium [11], this is the first study to our knowledge, to investigate the genes associated with decidualization and dissect out the local effects of trophoblast by using gestation matched ectopic and intrauterine pregnancies stratified for degree of decidualization. This has afforded unique insights not highlighted by previous studies and adds to our understanding of the endometrium in early pregnancy.

The crucial importance of decidualization in early pregnancy was highlighted in the this study as the gene expression profiles of the endometrium from patients clustered based on the degree of decidualization, irrespective of the location of the pregnancy. However, as expected [4] endometrium from ectopic pregnancies was less decidualized. We used the expression of *PRL* and *IGFBP1* as known markers of endometrial decidualization. These genes are universally accepted as markers of decidualization [4], [10]. When we grouped the endometrium from ectopic pregnancies on the degree of their histological decidualization, and assessed endometrium from ectopic pregnancies matched for decidualization with intrauterine pregnancies, the differential expression of *PRL* and *IGFBP1* supported these groups and our strategy for gene and pathway discovery.

Surprisingly *IGFBP1* was not highlighted in the array decidualization gene discovery study. Although the array showed a 20-fold increase in *IGFBP1* in the partially decidualized endometrium from ectopic pregnancies when compared to the non-decidualized samples, this difference failed on statistical test correction and was not included. We had previously showed that *INHBB* increased during decidualization and reduced expression could be a biomarker of EP [4]. Interestingly this gene was not identified in the decidualization studies but was shown to be increased by the presence of local trophoblast. Overall these examples highlight that although we have identified novel genes there may be additional differentially expressed important genes masked by the robust statistical analysis and that gene regulation may be complex and multiple approaches are required. However reassuringly these studies reinforced our previous discovery that *CRISP3* is a trophoblast-inhibited gene [5].

The success of our strategy is further highlighted by the discovery of a notable increase in *DKK1* with increasing decidualization. *DKK1* is known to be a marker of increasing decidualization [15], [16]. We showed that the gene with the highest fold change associated with endometrial decidual change detected in the microarray study was *SCARA5* and confirmed differential expression using quantitative RT-PCR. This gene has not previously been associated with decidualization and was not highlighted when ectopic decidua was compared to mid-secretory endometrium [11] or when endometrial stromal cells were decidualized *in vitro* [9]. It is of interest however, not only because of the marked differential expression, but because its expression is known to be altered by allelic imbalance or methylation [17], [18]. It is expressed by epithelial cells [19] and has been described in the bovine endometrial stroma particularly during pregnancy [20]. Nevertheless, the function of SCARA5 is not entirely clear. It has been postulated that it has a role in innate immune activity [19], [20] as well as lipid and ferritin trafficking [21]–[23]. It is likely that *SCARA5* has a role in the endometrial decidualization and highlights how potentially important novel genes may be detected by our approach.

*PROK1* is another gene highlighted by our approach that is also worthy of discussion. It is expressed in the endometrium and is increased in the secretory phase [24]. It has also been reported to be expressed in the decidua of early pregnancy and has been linked to the regulation of *COX2*, *LIF* and other genes known to be involved in implantation [25]. Although its secretion is promoted by local hCG administration [26], we have shown that it is related to decidualization in the absence of local trophoblast. Although it is related to decidualization it may not be regulated in the same way or at the same time as *PRL*. In recurrent pregnancy loss the mid-secretory endometrium has a decreased *PRL* expression but increased *PROK1* expression [27]. Our study has further emphasized the importance of this molecule and the need for further study.

*MMP7* was the gene whose expression was most down-regulated by decidualization of the endometrium. Matrix metalloproteinases (MMPs), including *MMP7* and *MMP11*, also found to be decreased in this study, are involved in degradation of the extracellular matrix (ECM) and control of MMPs during implantation is necessary for the maintenance of tissue stability in the uterus [28], [29]. This, and the up-regulation of the MMP inhibitor *TIMP3* found here, suggests that decidualization is associated with a suppression of genes involved in ECM remodelling and this occurs in the absence of local trophoblast. In their comparison of ectopic decidua with the mid-secretory endometrium, Savaris *et al.* reported *MMP7* and *MMP11* were the most up-regulated genes in ectopic decidua [11]. The discrepancy between this and our findings may be because the endometrium in ectopic pregnancy is less decidualized or because in pregnancy there is higher *MMP7* and *MMP11* expression than in the non-pregnant endometrium even although it is modified by decidualization. Interestingly, Sherwin *et al.* showed that exposure to hCG during simulated pregnancy in the secretory phase increased endometrial *MMP7* expression [3].

Other notable genes markedly down-regulated by decidualization, in the present study, were *SFRP4* and *SFRP1*. In the pregnant rat uterus, however, *SFRP4* expression was found to increase during decidualization and this was estrogen regulated [31]. This highlights species differences in the endometrial response to pregnancy. In contrast, in primates, a reduction in endometrial *SFRP4* was seen during hCG exposure of the endometrium *in vivo* [30]. The secreted frizzled-related proteins such as SFRP4 contain Wnt-binding domains and are soluble regulators of Wnt signalling pathways [31]. This would suggest that the Wnt pathway has a role in the promotion of decidualization. However, *DKK1* was markedly upregulated during decidualization. As DKK1 works by inhibiting the Wnt signalling pathway the role of Wnt in decidualization is complex. Interestingly, in the secretory compared to proliferative endometrium *DKK1* is upregulated while *SFRP4* is downregulated [32] suggesting co-ordinated inverse regulation of these pathways. In addition, *SFRP4* expression is also reduced in endometrial carcinomas [33] and sarcomas [34] confirming an important role in normal endometrial function.

However, the role of specific Wnts and their signalling pathways in the endometrium in early human pregnancy is not well understood [35]. This gap in knowledge is highlighted in the current study. We found the top canonical pathways involved in decidualization as natural killer cell (NK) signalling and Wnt-ß-catenin signalling. The importance of uterine NK cells in endometrial and decidual function is well known [10], [36], [37]. Further study of the complex Wnt system and its regulatory and signalling molecules in the human decidua is urgently required.

When endometrium from intrauterine and extrauterine pregnancies with matched decidualization status was examined, we were surprised by how few genes were significantly differentially expressed. Although trophoblast may have a direct paracrine effect on the neighboring endometrium, we have shown that differences in gene expression are modest. This would be consistent with the endometrium having a facilitory rather than dynamic response to pregnancy. However, as decidua could not be stratified by distance to implantation site this may imply that local effects of trophoblast are spatially limited in the endometrium and not seen in decidua more distant to the implantation site.

Four of the most up-regulated genes in the presence of local trophoblast, *CSH1*, *CSH2*, *CGA* and *CSHL1* are known trophoblast gene products [38], [39]. This would be consistent with possible trophoblast cell contamination not detected on initial immunohistochemical analysis. We confirmed however that non-trophoblast cells are able to express these chorionic related genes. We looked for *CSH1* and *CGA* transcripts in an epithelial cell line and found that, not only could they be detected, but there was a non-significant trend for their increase when exposed to physiologically relevant local concentrations of hCG [5]. This suggests that the endometrial expression of these genes may well by increased by trophoblast. If there was decidual microcontamination with trophoblast it is surprising how few genes were changed but more importantly it would further argue against significant global endometrial changes influenced by local trophoblast.

Another intriguing observation is that *CPA3*, *TPSAB1* and *TPSB2* were increased by local trophoblast. These are all mast cell products and this highlights another possible interaction of trophoblast with immune cells. It has been shown that mast cells are increased in choriocarcinoma and molar pregnancy and are thought to be involved in controlling trophoblast invasion [40]. The role of mast cells in successful pregnancy is unknown [41] but they have been linked to pregnancy failure [42]. A reduction in mast cells in ectopic pregnancy is unlikely as the cellular composition of uterine decidua in tubal and intrauterine pregnancies are similar [7], [8]. Some women in the matched decidualization intrauterine group had a non-viable pregnancy and, as pregnancy failure is associated with increased mast cells [41], [42], this may explain the difference. However even in this case it highlights the role of trophoblast in the regulation of decidual mast cells.

Although fewer genes were down-regulated by local trophoblast the top down-regulated gene has been described to be regulated by hCG. *CRISP3* is expressed in the endometrial epithelium and we have previously reported that its expression is increased in the decidua of ectopic pregnancy [5]. In addition *CRISP3* was reduced in ectopic decidua when compared to the mid-secretory endometrium [11]. *CRISP3* expression in an endometrial epithelial cell line was inhibited by physiological concentrations of hCG [5]. These data further support the concept that trophoblast can inhibit the expression of genes. *CRISP3* is widely expressed in the reproductive tract of males [43] and females [5], [44] with several functions attributed to it including a supporting role in localized tissue inflammation and immune response [45], [46]. It is therefore not surprising that pathway analysis confirmed pathways regulated by local trophoblast to include those involved in immune function and inflammation.

As well as *CRISP3* it seems that other molecules involved in the response to injury such as *UPK1B* are also down regulated by trophoblast. *UPKB1* was initially described in the bladder epithelium but has recently been reported in the glandular-epithelial cells of the endometrium [47]. It was increased after endometrial injury also suggesting a role in local inflammation. Indeed *GAST* is also up-regulated during inflammation [48], [49]. Although there may well be genes specifically inhibited by trophoblast it is notable that both *CRISP3* and *GAST* are also regulated by decidualization. This further highlights the fundamental importance of the decidualization process in early human pregnancy.

In conclusion, this study used a novel strategy to use tubal ectopic pregnancy to study genes associated with decidualization in the absence of local trophoblast. As well as confirming genes and pathways already implicated in endometrial decidual change, we have highlighted additional genes worthy of further study. In addition, we used decidualization matched tubal ectopic and intrauterine pregnancies to study the genetic effects of local trophoblast on the endometrium. This highlighted how few endometrial genes are regulated by trophoblast and further confirmed the fundamental importance of the decidualization process. We have shown that tubal ectopic pregnancy may be used to study decidualization and believe findings from this approach will further our understanding of the biology of embryo implantation and early pregnancy and its manipulation.

## Materials and Methods

### Tissue collection

Ethical approval for this study was obtained from Lothian Research Ethics Committee (04/S1103/20) and informed written consent was obtained from all patients before sample collection. Gestation-matched endometrium from IUP (57.25±1.9 days) and EP (58.75±0.9 days) were collected. IUP endometrium was obtained from women undergoing surgical management for termination of viable intrauterine pregnancy (n = 8) or missed miscarriage (n = 5). EP endometrium was obtained at the time of surgical management of tubal EP (n = 11), as described previously [4].

Each endometrial biopsy was divided into two pieces, one treated with RNAlater™ (Ambion, Austin, TX, USA) at 4 C overnight and then flash-frozen and stored at −70 C. The other piece was fixed in 10% neutral buffered formalin overnight at 4 C, transferred to 70% ethanol and embedded in paraffin wax. The absence of trophoblast contamination of the endometrial tissue was confirmed using immunohistochemical staining for Cytokeratin as described previously [50]. Tissue sections were also stained with haematoxylin and eosin for morphological analysis by an expert gynaecological histopathologist as described previously [4]. Briefly the decidualization status of each sample was blindly classified into three groups according to their degree of decidualization. Non-decidualized endometrium was classified as ‘−’ (n = 5), confluent decidual change was classified as ‘++’ (n = 7) and intermediate decidual change' was classified as ‘+’ (n = 12).

### RNA Extraction

Total RNA was extracted from the frozen endometrium using the ‘RNAeasy’ kit as detailed in the manufacturers' protocol (Qiagen, West Sussex, UK). The concentration and quality of the extracted RNA was assessed using an Agilent bioanalyser. All samples were standardized for quality control and assigned an RNA integrity number (RIN) [51] as described previously [4].

### Microarray Analysis

The conduct and robust standardization and assessment of the microarray studies using the Affymetrix platform have been previously reported [4]. Following data normalization by robust multiarray average (RMA) the data were filtered to remove invariant transcripts that would contribute to multiple testing errors in the subsequent statistical analysis, using an arbitrary signal intensity threshold value of 64. Hierarchical clustering analysis was performed on the filtered gene set using the Pearson Centroid method in Array Assist (Stratagene; Santa Rosa, CA, USA). Genes differentially expressed between groups were identified using a t-test comparison followed by multiple test correction using the Benjamini-Hochberg false discovery detection method [52]. Gene lists were then created using a fold change threshold of ≥2 and a corrected *P*-value of <0.05.

### Network identification and canonical pathway analysis

The microarray data were analysed using Ingenuity pathway analysis (IPA) software v4.0 (Ingenuity Systems, Redwood City, CA). Each gene identifier was mapped to its corresponding gene object in the Ingenuity pathways knowledge base (Ingenuity® Systems; www.ingenuity.com). Gene networks were then algorithmically generated based on their connectivity and assigned a score. The networks were then analyzed to identify the biological functions and/or diseases that were most significant to the genes in that network. Canonical pathway analysis identified the pathways that were most significant to the input data set. The significance of the association between the data set and the canonical pathway was determined based on two parameters: (i) a ratio of the number of genes from the data set that map to the pathway divided by the total number of genes that map to the canonical pathway and (ii) a *P*-value calculated using Fisher's exact test determining the probability that the association between the genes in the data set and the canonical pathway is due to chance alone.

### Cell culture

The endometrial epithelial cell line (hTERT-EEpC) [53] was maintained in culture as described previously [5]. After 24 h in serum-free conditions cells were treated in with physiological concentrations of hCG (Organon Lboratories, Cambridge, UK) as described previously [5]. After 24 h treatment RNA was extracted using the ‘RNAeasy’ kit following the manufacturer's instructions (Qiagen). This experiment was performed in triplicate.

### Quantitative RT-PCR

Quantitative RT-PCR was used to validate the array data for *PRL*, *IGFBP1*, *SCARA5*, *DKK1*, *MMP7*, *INHBA*, *CRISP3*, *GAST*, *CSH1*, *CGA*. RNA was reverse transcribed using Applied Biosystems reverse transcriptase (RT) kit (Applied Biosystems, UK) following the manufacturer's instructions. Taqman quantitative RT-PCR (qRT-PCR) was then used to measure mRNA levels using Applied Biosystems pre-validated ‘assay-on-demand’ primers and probes (www.universalprobelibrary.com) using a standard Taqman reaction mix and normalized against ribosomal 18S internal control primers and probe (Applied Biosystems). Samples were analyzed on an ABI Prism 7900 using standard conditions and differences quantified using the 2-ΔΔCt method as described previously [5].

### Statistical Analysis

All data were initially examined for normality of distribution using Prism 4 computer software (GraphPad Software; La Jolla, CA, USA). Where the data were normally distributed, groups were compared using a t-test and if not normally distributed, a non-parametric Mann-Whitney test was used. The cell culture experiments were analyzed using a non-parametric Kruskal Wallis test. Statistical significance was at the *P*<0.05 level and is indicated in the figure legend.

## Supporting Information

Table S1 List of all 658 genes with a FC of ≥2 derived from comparison of the array results from the decidua from women with ectopic pregnancies with little or no decidualization, and decidua from women with ectopic pregnancies with moderate decidualization.
(PDF)

Table S2 Top 20 canonical pathways associated with the array results comparing the decidua from women with ectopic pregnancies with little or no decidualization, and the decidua from women with ectopic pregnancies with moderate decidualization.
(PDF)

Table S3 List of all 85 genes with a FC≥2 derived from the comparison of the array results from decidua from women with ectopic pregnancies with moderate decidualization and decidua from women with intrauterine pregnancies with the same degree of decidualization.
(PDF)
