# Supplementary material for: Ectopic Pregnancy as a Model to Identify Endometrial Genes and Signaling Pathways Important in Decidualization and Regulated by Local Trophoblast
Source: PLoS One. 2011 Aug 17;6(8):e23595. doi: 10.1371/journal.pone.0023595 (PMC3157392; doi:10.1371/journal.pone.0023595)
Supplement: Table S1 — List of all 658 genes with a FC of ≥2 derived from comparison of the array results from the decidua from women with ectopic pregnancies with little or no decidualization, and decidua from women with ectopic pregnancies with moderate decidualization. (PDF) [file pone.0023595.s001.pdf]

Gene Symbol	Fold change	Regulation	Gene Title
MMP7	35.249332	down	matrix metallopeptidase 7 (matrilysin, uterine)
SFRP4	21.087008	down	secreted frizzled-related protein 4
SLC47A1	20.284035	down	solute carrier family 47, member 1
DIO2	17.768757	down	deiodinase, iodothyronine, type II
MMP11	16.544472	down	matrix metallopeptidase 11 (stromelysin 3)
CXCL2	14.970273	down	chemokine (C-X-C motif) ligand 2
INHBA	13.134676	down	inhibin, beta A
SERPINA1	13.030439	down	serpin peptidase inhibitor, clade A (alpha-1 antiproteinase, antitrypsin), member 1
TMSB15A	12.707266	down	thymosin beta 15a
MFAP2	12.560624	down	microfibrillar-associated protein 2
SFRP1	11.617197	down	secreted frizzled-related protein 1
MXRA5	11.211434	down	matrix-remodelling associated 5
LRRC17	10.844499	down	leucine rich repeat containing 17
PMEPA1	10.719318	down	prostate transmembrane protein, androgen induced 1
CNTN1	10.218915	down	Contactin 1
ANO1	8.601653	down	anoctamin 1, calcium activated chloride channel
IL7R	8.377788	down	interleukin 7 receptor
SDK2	8.104415	down	sidekick homolog 2 (chicken)
FGF9	7.5811973	down	fibroblast growth factor 9 (glia-activating factor)
CFTR	7.3389935	down	cystic fibrosis transmembrane conductance regulator (ATP-binding cassette sub-family C, member 7)
FJX1	6.42158	down	four jointed box 1 (Drosophila)
PAGE4	6.3176584	down	P antigen family, member 4 (prostate associated)
BMPR1B	6.1942167	down	bone morphogenetic protein receptor, type IB
BCL11A	6.1781006	down	B-cell CLL/lymphoma 11A (zinc finger protein)
TFPI2	6.162372	down	tissue factor pathway inhibitor 2
RASD1	6.1563287	down	RAS, dexamethasone-induced 1
PMAIP1	6.1341105	down	phorbol-12-myristate-13-acetate-induced protein 1
CRABP2	5.9918466	down	cellular retinoic acid binding protein 2
SOX17	5.9909754	down	SRY (sex determining region Y)-box 17
DPYSL4	5.8103333	down	dihydropyrimidinase-like 4
RGS4	5.768877	down	regulator of G-protein signaling 4
TMEM47	5.72366	down	transmembrane protein 47
ARNT2	5.7227683	down	aryl-hydrocarbon receptor nuclear translocator 2
ASRGL1	5.7183523	down	asparaginase like 1
GLT8D2	5.68125	down	glycosyltransferase 8 domain containing 2
FZD10	5.50634	down	frizzled homolog 10 (Drosophila)
PDGFC	5.4750986	down	platelet derived growth factor C
CPM	5.4378033	down	carboxypeptidase M
ADCYAP1R1	5.192887	down	Adenylate cyclase activating polypeptide 1 (pituitary) receptor type I
ZCCHC12	5.1830616	down	zinc finger, CCHC domain containing 12
ENC1	5.008188	down	ectodermal-neural cortex (with BTB-like domain)
SOX4	4.940779	down	SRY (sex determining region Y)-box 4
SCG5	4.9026165	down	secretogranin V (7B2 protein)
VANGL2	4.858861	down	vang-like 2 (van gogh, Drosophila)
TNC	4.817522	down	tenascin C
PRKCQ	4.8032594	down	protein kinase C, theta
AXIN2	4.739536	down	axin 2
CPA3	4.4433126	down	carboxypeptidase A3 (mast cell)
BICD1	4.4047527	down	bicaudal D homolog 1 (Drosophila)
ESR1	4.393155	down	estrogen receptor 1
WFDC2	4.348775	down	WAP four-disulfide core domain 2
NTN1	4.341155	down	netrin 1
ISM1	4.331676	down	isthmin 1 homolog (zebrafish)
BGN	4.331442	down	biglycan
DUSP4	4.3129115	down	dual specificity phosphatase 4
ARL4C	4.2967105	down	ADP-ribosylation factor-like 4C
CAMK2N1	4.2953506	down	calcium/calmodulin-dependent protein kinase II inhibitor 1
CXCL1	4.248615	down	chemokine (C-X-C motif) ligand 1 (melanoma growth stimulating activity, alpha)
BNC2	4.1695466	down	basonuclin 2
BEX2	4.0682435	down	brain expressed X-linked 2
NLRP1	4.0667086	down	NLR family, pyrin domain containing 1
PGM2L1	4.0146236	down	phosphoglucomutase 2-like 1
ETV1	3.973477	down	ets variant 1
RNF183	3.8828452	down	ring finger protein 183
PLCB1	3.8785582	down	phospholipase C, beta 1 (phosphoinositide-specific)
CAPN6	3.8432379	down	calpain 6
DNM1	3.8406756	down	dynamin 1
NAV2	3.8373709	down	neuron navigator 2
MAP2K6	3.8014016	down	mitogen-activated protein kinase kinase 6
ZNF185	3.8012047	down	zinc finger protein 185 (LIM domain)
CDH2	3.7871976	down	cadherin 2, type 1, N-cadherin (neuronal)
FRAS1	3.7129247	down	Fraser syndrome 1
ID1	3.6626325	down	inhibitor of DNA binding 1, dominant negative helix-loop-helix protein
MSX1	3.6508365	down	msh homeobox 1
DYNC1I1	3.6447763	down	dynein, cytoplasmic 1, intermediate chain 1
EPHA7	3.5860841	down	EPH receptor A7
ELF3	3.5777204	down	E74-like factor 3 (ets domain transcription factor, epithelial-specific)
CLIC6	3.5618336	down	chloride intracellular channel 6
PGRMC1	3.5608792	down	progesterone receptor membrane component 1
WNT2	3.5432081	down	wingless-type MMTV integration site family member 2
LOXL1	3.5089707	down	lysyl oxidase-like 1
COL7A1	3.4833744	down	collagen, type VII, alpha 1
RBM24	3.4650342	down	RNA binding motif protein 24
BHLHE41	3.4450722	down	basic helix-loop-helix family, member e41
SLC26A2	3.4245265	down	solute carrier family 26 (sulfate transporter), member 2
MEX3D	3.4077044	down	mex-3 homolog D (C. elegans)
SALL2	3.4004586	down	sal-like 2 (Drosophila)
NEDD9	3.396467	down	neural precursor cell expressed, developmentally down-regulated 9
PCSK5	3.3921711	down	Proprotein convertase subtilisin/kexin type 5
CYTSB	3.3801382	down	cytospin B
ANK3	3.3515372	down	ankyrin 3, node of Ranvier (ankyrin G)
SYT1	3.3081925	down	synaptotagmin I
EDIL3	3.2711883	down	EGF-like repeats and discoidin I-like domains 3
AGR3	3.2521636	down	anterior gradient homolog 3 (Xenopus laevis)

GABRP	3.2316995	down	gamma-aminobutyric acid (GABA) A receptor, pi
ENAH	3.1090417	down	enabled homolog (Drosophila)
TPSAB1	3.0931718	down	tryptase alpha/beta 1
BMF	3.082947	down	Bcl2 modifying factor
WNT5A	3.0572298	down	wingless-type MMTV integration site family, member 5A
PALLD	3.0216181	down	palladin, cytoskeletal associated protein
OLFML2A	3.0048687	down	olfactomedin-like 2A
CACNA1G	2.9903586	down	calcium channel, voltage-dependent, T type, alpha 1G subunit
DENND2A	2.9766312	down	DENN/MADD domain containing 2A
TBX3	2.939192	down	T-box 3
TCF4	2.9302144	down	transcription factor 4
PELI2	2.9261239	down	pellino homolog 2 (Drosophila)
TRIB2	2.9259903	down	tribbles homolog 2 (Drosophila)
SALL1	2.9202485	down	sal-like 1 (Drosophila)
CA11	2.9162571	down	carbonic anhydrase XI
SMAD7	2.8881986	down	SMAD family member 7
LTBP1	2.8774908	down	latent transforming growth factor beta binding protein 1
TPBG	2.8765852	down	trophoblast glycoprotein
JUB	2.8742502	down	jub, ajuba homolog (Xenopus laevis)
NT5DC2	2.8602989	down	5'-nucleotidase domain containing 2
NBL1	2.8476171	down	neuroblastoma, suppression of tumorigenicity 1
APOL4	2.8363001	down	apolipoprotein L, 4
PHC1	2.7991002	down	polyhomeotic homolog 1 (Drosophila)
CPSF6	2.79436	down	cleavage and polyadenylation specific factor 6, 68kDa
TEAD2	2.7741835	down	TEA domain family member 2
CARD10	2.7662368	down	caspase recruitment domain family, member 10
GALNT4	2.7602742	down	UDP-N-acetyl-alpha-D-galactosamine:polypeptide N-acetylgalactosaminyltransferase 4 (GalNAc-T4)
SLC7A1	2.7521625	down	solute carrier family 7 (cationic amino acid transporter, y+ system), member 1
TRPM6	2.7463026	down	Transient receptor potential cation channel, subfamily M, member 6
TMEM132A	2.7307737	down	transmembrane protein 132A
ZCCHC14	2.711077	down	zinc finger, CCHC domain containing 14
IRS1	2.6954856	down	insulin receptor substrate 1
SORBS2	2.6818519	down	sorbin and SH3 domain containing 2
KLHL14	2.6767123	down	kelch-like 14 (Drosophila)
FZD2	2.673267	down	frizzled homolog 2 (Drosophila)
BEX1	2.6625004	down	brain expressed, X-linked 1
MAP2	2.6572318	down	microtubule-associated protein 2
GATA2	2.6571105	down	GATA binding protein 2
ZNF323	2.6540935	down	zinc finger protein 323
CCDC74A /// CCDC74B	2.6459951	down	coiled-coil domain containing 74A /// coiled-coil domain containing 74B
OCIAD2	2.6232836	down	OCIA domain containing 2
MAGED4 /// MAGED4B	2.6088336	down	melanoma antigen family D, 4 /// melanoma antigen family D, 4B
DACH1	2.5760481	down	dachshund homolog 1 (Drosophila)
SETBP1	2.5724773	down	SET binding protein 1
TSC22D1	2.5627642	down	TSC22 domain family, member 1
AR	2.5480807	down	androgen receptor
CXADR	2.5319421	down	coxsackie virus and adenovirus receptor
HGF	2.5263412	down	hepatocyte growth factor (hepapoietin A; scatter factor)
RAD54B	2.5250459	down	RAD54 homolog B (S. cerevisiae)
DKK3	2.5106943	down	dickkopf homolog 3 (Xenopus laevis)
ZNF423	2.498062	down	zinc finger protein 423
CSRP2	2.4963071	down	cysteine and glycine-rich protein 2
NET1	2.4955585	down	neuroepithelial cell transforming 1
CEP70	2.487023	down	centrosomal protein 70kDa
GPR125	2.484246	down	G protein-coupled receptor 125
RNF144A	2.4577322	down	ring finger protein 144A
MSI2	2.4514372	down	musashi homolog 2 (Drosophila)
GABRB3	2.445523	down	gamma-aminobutyric acid (GABA) A receptor, beta 3
ISYNA1	2.4366891	down	inositol-3-phosphate synthase 1
HNRNPR	2.4349816	down	heterogeneous nuclear ribonucleoprotein R
KDM6B	2.4134023	down	lysine (K)-specific demethylase 6B
MPZL2	2.4091833	down	myelin protein zero-like 2
PORCN	2.4075873	down	porcupine homolog (Drosophila)
IKZF2	2.407297	down	IKAROS family zinc finger 2 (Helios)
NRIP1	2.4003637	down	nuclear receptor interacting protein 1
FAM69B	2.3997862	down	family with sequence similarity 69, member B
BEND5	2.3855402	down	BEN domain containing 5
PHC1 /// PHC1B	2.3775709	down	polyhomeotic homolog 1 (Drosophila) /// polyhomeotic homolog 1B (Drosophila)
PRRX1	2.371472	down	paired related homeobox 1
ANKRD50	2.3707454	down	ankyrin repeat domain 50
ZNF532	2.3706036	down	zinc finger protein 532
ALDH2	2.3583546	down	aldehyde dehydrogenase 2 family (mitochondrial)
CLIC5	2.3547082	down	chloride intracellular channel 5
GDF11	2.3522067	down	growth differentiation factor 11
BEND7	2.3367698	down	BEN domain containing 7
SYT11	2.3321419	down	synaptotagmin XI
ZNF300	2.3113472	down	zinc finger protein 300
MDK	2.3026052	down	midkine (neurite growth-promoting factor 2)
H2BFM /// H2BFXP	2.302238	down	H2B histone family, member M /// H2B histone family, member X, pseudogene
ANAPC4	2.2940674	down	anaphase promoting complex subunit 4
ADRBK2	2.2934005	down	adrenergic, beta, receptor kinase 2
EVI1	2.290167	down	ecotropic viral integration site 1
KIF5C	2.2847998	down	kinesin family member 5C
SNCAIP	2.2831664	down	synuclein, alpha interacting protein
TSPAN11	2.2707267	down	tetraspanin 11
TGIF2	2.2532291	down	TGFB-induced factor homeobox 2
CACNA1A	2.2520416	down	calcium channel, voltage-dependent, P/Q type, alpha 1A subunit
MEX3B	2.243988	down	mex-3 homolog B (C. elegans)
PLK2	2.241044	down	polo-like kinase 2 (Drosophila)
KCTD1	2.2409132	down	potassium channel tetramerisation domain containing 1
ZNF117	2.2371233	down	zinc finger protein 117
SBK1	2.2289643	down	SH3-binding domain kinase 1
MYLK	2.2230575	down	myosin light chain kinase
L3MBTL3	2.219215	down	l(3)mbt-like 3 (Drosophila)
ALCAM	2.2170587	down	activated leukocyte cell adhesion molecule

RASSF8	2.2128198	down	Ras association (RalGDS/AF-6) domain family (N-terminal) member 8
EHF	2.209567	down	Ets homologous factor
EPPK1	2.2088478	down	epiplakin 1
KRT8	2.2016246	down	keratin 8
TGIF1	2.1859207	down	TGFB-induced factor homeobox 1
CHST6	2.1832507	down	carbohydrate (N-acetylglucosamine 6-O) sulfotransferase 6
SIPA1L1	2.1773477	down	Signal-induced proliferation-associated 1 like 1
USP31	2.1747463	down	ubiquitin specific peptidase 31
RARB	2.1732068	down	retinoic acid receptor, beta
KRT17	2.1700816	down	keratin 17
TCF3	2.1659224	down	transcription factor 3 (E2A immunoglobulin enhancer binding factors E12/E47)
DLG3	2.1657116	down	discs, large homolog 3 (Drosophila)
SNRPN	2.1552086	down	small nuclear ribonucleoprotein polypeptide N
HMGB3	2.154053	down	high-mobility group box 3
PAX2	2.1508896	down	paired box 2
MEX3A	2.135047	down	mex-3 homolog A (C. elegans)
hCG_2024094	2.1349802	down	hCG2024094
SERTAD4	2.1326706	down	SERTA domain containing 4
SLC25A37	2.1320355	down	solute carrier family 25, member 37
DACT3	2.1172135	down	dapper, antagonist of beta-catenin, homolog 3 (Xenopus laevis)
SDS	2.1139412	down	serine dehydratase
NEO1	2.113462	down	neogenin homolog 1 (chicken)
KLHL23	2.1018033	down	kelch-like 23 (Drosophila)
PURG	2.0790863	down	purine-rich element binding protein G
CTPS2	2.0779092	down	CTP synthase II
PFKFB3	2.072646	down	6-phosphofructo-2-kinase/fructose-2,6-biphosphatase 3
SPIN1	2.0557718	down	spindlin 1
LRCH2	2.0535543	down	leucine-rich repeats and calponin homology (CH) domain containing 2
CACHD1	2.0452394	down	cache domain containing 1
ATXN1	2.0408626	down	ataxin 1
TSPAN6	2.039551	down	tetraspanin 6
NES	2.037787	down	nestin
LEF1	2.037775	down	lymphoid enhancer-binding factor 1
HOXB3	2.0289664	down	homeobox B3
SSX2IP	2.0179725	down	synovial sarcoma, X breakpoint 2 interacting protein
SLFN5	2.0134792	down	schlafen family member 5
VEZF1	2.0129452	down	vascular endothelial zinc finger 1
NASP	2.0112748	down	nuclear autoantigenic sperm protein (histone-binding)
GALNT10	2.0086548	down	UDP-N-acetyl-alpha-D-galactosamine:polypeptide N-acetylgalactosaminyltransferase 10 (GalNAc-T10)
IPW	2.0068834	down	imprinted in Prader-Willi syndrome (non-protein coding)
TPSAB1 /// TPSB2	2.0000558	down	tryptase alpha/beta 1 /// tryptase beta 2
SCARA5	181.89972	up	Scavenger receptor class A, member 5 (putative)
DKK1	70.708755	up	dickkopf homolog 1 (Xenopus laevis)
PRL	47.057854	up	prolactin
CHRDL1	31.622993	up	chordin-like 1
PROK1	18.936087	up	prokineticin 1
ANKRD55	17.513077	up	Ankyrin repeat domain 55
TIMP3	17.02266	up	TIMP metallopeptidase inhibitor 3
ST6GALNAC5	15.460088	up	ST6 (alpha-N-acetyl-neuraminyl-2,3-beta-galactosyl-1,3)-N-acetylgalactosaminide alpha-2,6-sialyltransferase 5
COLEC11	14.460702	up	collectin sub-family member 11
CFD	14.153963	up	complement factor D (adipsin)
GAST	14.122097	up	gastrin
APOD	13.97643	up	apolipoprotein D
CYP2C9	13.954113	up	cytochrome P450, family 2, subfamily C, polypeptide 9
SLC18A2	13.743943	up	solute carrier family 18 (vesicular monoamine), member 2
AOX1	13.5775175	up	aldehyde oxidase 1
FGF7 /// KGFLP1 /// KGFLP2	13.456108	up	fibroblast growth factor 7 (keratinocyte growth factor) /// keratinocyte growth factor-like protein 1,2
NAV3	13.174094	up	neuron navigator 3
CYP4B1	12.787078	up	cytochrome P450, family 4, subfamily B, polypeptide 1
ALDH8A1	12.761514	up	aldehyde dehydrogenase 8 family, member A1
ANGPTL1	12.678688	up	angiopoietin-like 1
CNR1	12.047418	up	cannabinoid receptor 1 (brain)
GPC3	11.909819	up	glypican 3
ADAMTS5	11.5914135	up	ADAM metallopeptidase with thrombospondin type 1 motif, 5
FAM5B	11.454284	up	family with sequence similarity 5, member B
MT1M	11.372846	up	metallothionein 1M
ALOX12	11.267488	up	arachidonate 12-lipoxygenase
GABRA2	11.074262	up	gamma-aminobutyric acid (GABA) A receptor, alpha 2
GALNT13	10.801902	up	UDP-N-acetyl-alpha-D-galactosamine:polypeptide N-acetylgalactosaminyltransferase 13 (GalNAc-T13)
KIR2DS1 /// KIR2DS2 /// KIR2DS4	10.638587	up	killer cell immunoglobulin-like receptor, two domains, short cytoplasmic tail, 1,2,4
SLC44A1	9.606816	up	solute carrier family 44, member 1
COMP	9.492166	up	cartilage oligomeric matrix protein
KIR2DL4	9.127718	up	killer cell immunoglobulin-like receptor, two domains, long cytoplasmic tail, 4
IL15	8.868091	up	interleukin 15
KIR3DL2	8.60016	up	killer cell immunoglobulin-like receptor, three domains, long cytoplasmic tail, 2
KIR2DL3	8.277727	up	killer cell immunoglobulin-like receptor, two domains, long cytoplasmic tail, 3
TRPC4	8.245244	up	transient receptor potential cation channel, subfamily C, member 4
DIRAS2	8.187207	up	DIRAS family, GTP-binding RAS-like 2
OMD	8.064551	up	osteomodulin
FGF7	7.99083	up	fibroblast growth factor 7 (keratinocyte growth factor)
KIR3DL1	7.92878	up	killer cell immunoglobulin-like receptor, three domains, long cytoplasmic tail, 1
MUTYH	7.8123493	up	mutY homolog (E. coli)
CHST2	7.7864265	up	carbohydrate (N-acetylglucosamine-6-O) sulfotransferase 2
MAOA	7.4372597	up	monoamine oxidase A
GPBAR1	7.3815427	up	G protein-coupled bile acid receptor 1
KAL1	7.2932296	up	Kallmann syndrome 1 sequence
RASGRP2	7.2387114	up	RAS guanyl releasing protein 2 (calcium and DAG-regulated)
ABAT	7.238233	up	4-aminobutyrate aminotransferase
GRIA3	7.199948	up	glutamate receptor, ionotrophic, AMPA 3
GATA6	7.1767225	up	GATA binding protein 6
KIR3DL3	6.997669	up	killer cell immunoglobulin-like receptor, three domains, long cytoplasmic tail, 3
CATSPERB	6.9503093	up	cation channel, sperm-associated, beta
NOV	6.9262486	up	nephroblastoma overexpressed gene
PILRA	6.9166784	up	paired immunoglobin-like type 2 receptor alpha
CTSW	6.8493423	up	cathepsin W

CITED2	6.8364472	up	Cbp/p300-interacting transactivator, with Glu/Asp-rich carboxy-terminal domain, 2
KLRC1 /// KLRC2	6.816638	up	killer cell lectin-like receptor subfamily C, member 1,2
KIR2DL2	6.703643	up	killer cell immunoglobulin-like receptor, two domains, long cytoplasmic tail, 2
GABRQ	6.630265	up	gamma-aminobutyric acid (GABA) receptor, theta
CHODL	6.5773606	up	chondrolectin
TMEM27	6.528342	up	transmembrane protein 27
ATP2C2	6.506031	up	ATPase, Ca++ transporting, type 2C, member 2
VCAM1	6.5028677	up	vascular cell adhesion molecule 1
NCAM1	6.433451	up	neural cell adhesion molecule 1
GLB1L2	6.418861	up	galactosidase, beta 1-like 2
MUC15	6.3632607	up	mucin 15, cell surface associated
ALPK2	6.323234	up	alpha-kinase 2
ENPEP	6.277534	up	glutamyl aminopeptidase (aminopeptidase A)
STAP1	6.257253	up	signal transducing adaptor family member 1
EMILIN2	6.1584096	up	elastin microfibril interfacer 2
TRA@ /// TRD@	6.14383	up	T cell receptor alpha locus /// T cell receptor delta locus
KIR2DL1	6.0698404	up	killer cell immunoglobulin-like receptor, two domains, long cytoplasmic tail, 1
ADRA2A	6.0355167	up	adrenergic, alpha-2A-, receptor
IL1R2	5.8638287	up	interleukin 1 receptor, type II
NPR1	5.7391386	up	natriuretic peptide receptor A/guanylate cyclase A (atrionatriuretic peptide receptor A)
HIST1H1C	5.7245727	up	histone cluster 1, H1c
KIR3DS1	5.7032013	up	killer cell immunoglobulin-like receptor, three domains, short cytoplasmic tail, 1
ULK4	5.6330256	up	unc-51-like kinase 4 (C. elegans)
NOG	5.618685	up	noggin
NTRK2	5.574129	up	neurotrophic tyrosine kinase, receptor, type 2
CACNB2	5.5483937	up	calcium channel, voltage-dependent, beta 2 subunit
HS2ST1	5.4257503	up	heparan sulfate 2-O-sulfotransferase 1
STYK1	5.4139004	up	serine/threonine/tyrosine kinase 1
SNX10	5.3736563	up	sorting nexin 10
KIR2DS3	5.3514185	up	killer cell immunoglobulin-like receptor, two domains, short cytoplasmic tail, 3
KIR2DS1	5.3495855	up	killer cell immunoglobulin-like receptor, two domains, short cytoplasmic tail, 1
PC	5.342878	up	pyruvate carboxylase
SLC3A1	5.2714577	up	solute carrier family 3, member 1
CRYAB	5.263656	up	crystallin, alpha B
WNT5B	5.2265596	up	wingless-type MMTV integration site family, member 5B
CYP11A1	5.2178407	up	cytochrome P450, family 11, subfamily A, polypeptide 1
UBASH3B	5.2081017	up	ubiquitin associated and SH3 domain containing, B
EDNRB	5.178998	up	endothelin receptor type B
TRD@	5.1578526	up	T cell receptor delta locus
FAM89A	5.139919	up	family with sequence similarity 89, member A
TMEM45A	5.1392846	up	transmembrane protein 45A
CDC7	5.136491	up	cell division cycle 7 homolog (S. cerevisiae)
DTNA	5.096747	up	dystrobrevin, alpha
ACADSB	5.0895915	up	acyl-Coenzyme A dehydrogenase, short/branched chain
KIR2DS5	5.0745816	up	killer cell immunoglobulin-like receptor, two domains, short cytoplasmic tail, 5
GZMA	5.0562706	up	granzyme A (granzyme 1, cytotoxic T-lymphocyte-associated serine esterase 3)
PLCL1	5.055071	up	phospholipase C-like 1
GNLY	5.0045156	up	granulysin
HSPB6	4.990287	up	heat shock protein, alpha-crystallin-related, B6
PI15	4.9567857	up	peptidase inhibitor 15
NKG7	4.92169	up	natural killer cell group 7 sequence
EOMES	4.886217	up	eomesodermin homolog (Xenopus laevis)
KIR2DL5A	4.879538	up	killer cell immunoglobulin-like receptor, two domains, long cytoplasmic tail, 5A
SLIT1	4.8473153	up	slit homolog 1 (Drosophila)
PGDS	4.8028054	up	prostaglandin D2 synthase, hematopoietic
PRUNE2	4.7991495	up	prune homolog 2 (Drosophila)
MAMDC2	4.7973886	up	MAM domain containing 2
ITGAD	4.745359	up	integrin, alpha D
SH2D1B	4.7323093	up	SH2 domain containing 1B
PAIP2B	4.724874	up	poly(A) binding protein interacting protein 2B
MTF1	4.707397	up	metal-regulatory transcription factor 1
DOCK8	4.70479	up	dedicator of cytokinesis 8
GAS1	4.65965	up	growth arrest-specific 1
SAMD3	4.651084	up	sterile alpha motif domain containing 3
PROCR	4.6428943	up	protein C receptor, endothelial (EPCR)
PALMD	4.6137567	up	palmdelphin
BCAT1	4.5960603	up	branched chain aminotransferase 1, cytosolic
NBLA00301	4.590337	up	Nbla00301
FAM69A	4.587559	up	family with sequence similarity 69, member A
KLRD1	4.5128217	up	killer cell lectin-like receptor subfamily D, member 1
MT1G	4.5102234	up	metallothionein 1G
RAB7B	4.4696245	up	RAB7B, member RAS oncogene family
IGFBP2	4.42673	up	insulin-like growth factor binding protein 2, 36kDa
C2	4.4193134	up	complement component 2
PLAC9	4.334226	up	placenta-specific 9
SLCO4C1	4.329807	up	solute carrier organic anion transporter family, member 4C1
ST3GAL5	4.3277435	up	ST3 beta-galactoside alpha-2,3-sialyltransferase 5
HTR2A	4.3225317	up	5-hydroxytryptamine (serotonin) receptor 2A
LINGO2	4.2987666	up	leucine rich repeat and Ig domain containing 2
CORO2A	4.2488513	up	coronin, actin binding protein, 2A
ADAMTSL4	4.2418237	up	ADAMTS-like 4
MT1F	4.23803	up	metallothionein 1F
ATP8B4	4.23123	up	ATPase, class I, type 8B, member 4
LMCD1	4.2168636	up	LIM and cysteine-rich domains 1
ABI3BP	4.211492	up	ABI family, member 3 (NESH) binding protein
MGST1	4.20139	up	microsomal glutathione S-transferase 1
ADORA3	4.1802115	up	adenosine A3 receptor
ERP27	4.148997	up	endoplasmic reticulum protein 27
KALRN	4.1436114	up	kalirin, RhoGEF kinase
LRIG3	4.134646	up	leucine-rich repeats and immunoglobulin-like domains 3
SFTA2	4.1199074	up	surfactant associated 2
TRNP1	4.1070666	up	TMF1-regulated nuclear protein 1
LYPLAL1	4.077351	up	lysophospholipase-like 1
MT1E	4.0697246	up	metallothionein 1E
IP6K3	4.04747	up	inositol hexakisphosphate kinase 3

APOA1	4.038557	up	apolipoprotein A-I
GP1BB /// SEPT5	3.927015	up	glycoprotein Ib (platelet), beta polypeptide /// septin 5
TBXA2R	3.906783	up	thromboxane A2 receptor
MT1H	3.901146	up	metallothionein 1H
IL2RB	3.892662	up	interleukin 2 receptor, beta
IRS2	3.886441	up	insulin receptor substrate 2
PHYHIPL	3.8659573	up	phytanoyl-CoA 2-hydroxylase interacting protein-like
GGT1	3.8651288	up	gamma-glutamyltransferase 1
PRF1	3.8253431	up	perforin 1 (pore forming protein)
TMEM37	3.8158991	up	transmembrane protein 37
RAB27B	3.8011239	up	RAB27B, member RAS oncogene family
ENPP1	3.7973995	up	ectonucleotide pyrophosphatase/phosphodiesterase 1
FGGY	3.6958861	up	FGGY carbohydrate kinase domain containing
GZMB	3.6932614	up	granzyme B (granzyme 2, cytotoxic T-lymphocyte-associated serine esterase 1)
CD96	3.6615925	up	CD96 molecule
SLC30A2	3.6428897	up	solute carrier family 30 (zinc transporter), member 2
ENTPD3	3.6329267	up	ectonucleoside triphosphate diphosphohydrolase 3
HSPB2	3.5721862	up	heat shock 27kDa protein 2
S100A4	3.5465899	up	S100 calcium binding protein A4
GLRX	3.5105052	up	glutaredoxin (thioltransferase)
CRISP3	3.5073345	up	cysteine-rich secretory protein 3
KLRC3	3.499376	up	killer cell lectin-like receptor subfamily C, member 3
PYHIN1	3.4814456	up	pyrin and HIN domain family, member 1
FAM134B	3.479267	up	family with sequence similarity 134, member B
MT1E /// MT1H /// MT1M /// MT1P2	3.4555683	up	metallothionein 1E /// metallothionein 1H /// metallothionein 1M /// metallothionein 1 pseudogene 2
OBFC2A	3.4454432	up	oligonucleotide/oligosaccharide-binding fold containing 2A
SLC12A8	3.4374342	up	solute carrier family 12 (potassium/chloride transporters), member 8
RSPO3	3.4287581	up	R-spondin 3 homolog (Xenopus laevis)
FOLR2	3.4242864	up	folate receptor 2 (fetal)
CSF2RA	3.4007614	up	colony stimulating factor 2 receptor, alpha, low-affinity (granulocyte-macrophage)
LYN	3.3961697	up	v-yes-1 Yamaguchi sarcoma viral related oncogene homolog
ACSM3	3.3947654	up	acyl-CoA synthetase medium-chain family member 3
MT1P2	3.3770492	up	metallothionein 1 pseudogene 2
GGT1 ///2 /// GGT3P /// GGTLC2 ///LC3	3.3656535	up	gamma-glutamyltransferase 1 /// 2 /// 3 pseudogene /// light chain 2 /// light chain 3
GPR126	3.3618581	up	G protein-coupled receptor 126
CORO1A	3.357142	up	coronin, actin binding protein, 1A
CXCL14	3.345366	up	chemokine (C-X-C motif) ligand 14
RHOQ	3.3414793	up	ras homolog gene family, member Q
RNASE1	3.3251963	up	ribonuclease, RNase A family, 1 (pancreatic)
MAOB	3.324438	up	monoamine oxidase B
RAB2A	3.3186514	up	RAB2A, member RAS oncogene family
FERMT2	3.317596	up	fermitin family homolog 2 (Drosophila)
RGS22	3.306687	up	regulator of G-protein signaling 22
MT1X	3.295584	up	metallothionein 1X
KCND3	3.283069	up	potassium voltage-gated channel, Shal-related subfamily, member 3
PLA2G16	3.2788942	up	phospholipase A2, group XVI
OSBPL10	3.2392266	up	oxysterol binding protein-like 10
PVRIG	3.2357173	up	poliovirus receptor related immunoglobulin domain containing
GPR82	3.234287	up	G protein-coupled receptor 82
FBLN2	3.2284431	up	fibulin 2
PAPSS2	3.2119427	up	3'-phosphoadenosine 5'-phosphosulfate synthase 2
GMNN	3.2103	up	geminin, DNA replication inhibitor
UGT1A1 ///A10 ///A4 ///A6 ///A8 ///A9	3.2028883	up	UDP glucuronosyltransferase 1 family, polypeptide A1 /// A10 /// A4 /// A6 /// A8 /// A9
DPH3	3.1953092	up	DPH3, KTI11 homolog (S. cerevisiae)
AFAP1L2	3.164219	up	actin filament associated protein 1-like 2
CYP26A1	3.1580567	up	cytochrome P450, family 26, subfamily A, polypeptide 1
TWISTNB	3.13846	up	TWIST neighbor
CCDC68	3.13806	up	coiled-coil domain containing 68
SLC45A4	3.1120617	up	solute carrier family 45, member 4
CHST7	3.106893	up	carbohydrate (N-acetylglucosamine 6-O) sulfotransferase 7
GNAI1	3.0964837	up	guanine nucleotide binding protein (G protein), alpha inhibiting activity polypeptide 1
FASLG	3.0884562	up	Fas ligand (TNF superfamily, member 6)
CORO2B	3.0871863	up	coronin, actin binding protein, 2B
STAT4	3.085296	up	signal transducer and activator of transcription 4
OLIG3	3.0833604	up	oligodendrocyte transcription factor 3
FAM49A	3.0628858	up	family with sequence similarity 49, member A
SLA2	3.060513	up	Src-like-adaptor 2
FAM46C	3.0575175	up	family with sequence similarity 46, member C
LASS6	3.047198	up	LAG1 homolog, ceramide synthase 6
KREMEN1	3.04032	up	kringle containing transmembrane protein 1
S100A3	3.032361	up	S100 calcium binding protein A3
NUPR1	3.0259175	up	nuclear protein 1
VPS13D	3.0101001	up	vacuolar protein sorting 13 homolog D (S. cerevisiae)
SPOCK1	2.9842224	up	sparc/osteonectin, cwcv and kazal-like domains proteoglycan (testican) 1
PAK3	2.9745603	up	p21 protein (Cdc42/Rac)-activated kinase 3
TXK	2.9725788	up	TXK tyrosine kinase
STARD5	2.9616928	up	StAR-related lipid transfer (START) domain containing 5
ACE2	2.9559338	up	angiotensin I converting enzyme (peptidyl-dipeptidase A) 2
ADCY1	2.9539423	up	adenylate cyclase 1 (brain)
TSPAN12	2.952221	up	tetraspanin 12
SNX21	2.9491277	up	sorting nexin family member 21
TGM2	2.9477859	up	transglutaminase 2 (C polypeptide, protein-glutamine-gamma-glutamyltransferase)
CAV2	2.943992	up	caveolin 2
DNAJB9	2.939391	up	DnaJ (Hsp40) homolog, subfamily B, member 9
GGTLC1	2.935339	up	gamma-glutamyltransferase light chain 1
JPH1	2.934403	up	junctophilin 1
GPD1L	2.9210181	up	glycerol-3-phosphate dehydrogenase 1-like
HPSE	2.9172623	up	heparanase
ALDH3B1	2.904439	up	aldehyde dehydrogenase 3 family, member B1
RAPH1	2.9042976	up	Ras association (RalGDS/AF-6) and pleckstrin homology domains 1
BOC	2.900468	up	Boc homolog (mouse)
HIST1H2BD	2.898394	up	histone cluster 1, H2bd
BTBD3	2.897809	up	BTB (POZ) domain containing 3
ARHGAP9	2.896556	up	Rho GTPase activating protein 9
ABLIM3	2.8939977	up	actin binding LIM protein family, member 3

RHOU	2.889771	up	ras homolog gene family, member U
EIF4E3	2.8798578	up	eukaryotic translation initiation factor 4E family member 3
CALHM2	2.8672636	up	calcium homeostasis modulator 2
LCP1	2.8509238	up	lymphocyte cytosolic protein 1 (L-plastin)
ITM2A	2.8505917	up	integral membrane protein 2A
STON1	2.8499057	up	stonin 1
ARHGAP24	2.8336916	up	Rho GTPase activating protein 24
GPR176	2.8124917	up	G protein-coupled receptor 176
DCN	2.8017433	up	decorin
HYAL1	2.7943919	up	hyaluronoglucosaminidase 1
PDLIM2	2.7896106	up	PDZ and LIM domain 2 (mystique)
CD247	2.7814705	up	CD247 molecule
ARHGEF6	2.77165	up	Rac/Cdc42 guanine nucleotide exchange factor (GEF) 6
TMTC1	2.770676	up	transmembrane and tetratricopeptide repeat containing 1
MCC	2.7624147	up	mutated in colorectal cancers
CFL2	2.7518365	up	cofilin 2 (muscle)
PSTPIP1	2.7206533	up	proline-serine-threonine phosphatase interacting protein 1
HIST1H2BK	2.7127898	up	histone cluster 1, H2bk
APOBEC3G	2.7119386	up	apolipoprotein B mRNA editing enzyme, catalytic polypeptide-like 3G
AKAP13	2.6742148	up	A kinase (PRKA) anchor protein 13
C1QTNF1	2.6717966	up	C1q and tumor necrosis factor related protein 1
KIAA1644	2.669179	up	KIAA1644
SLC25A19	2.6490548	up	solute carrier family 25 (mitochondrial thiamine pyrophosphate carrier), member 19
H2BFS	2.645481	up	H2B histone family, member S
DLGAP1	2.6409953	up	discs, large (Drosophila) homolog-associated protein 1 /// hypothetical protein LOC284214
HOPX	2.6377416	up	HOP homeobox
APOBEC3F /// APOBEC3G	2.6359596	up	apolipoprotein B mRNA editing enzyme, catalytic polypeptide-like 3F /// 3G
SASH3	2.6353185	up	SAM and SH3 domain containing 3
OSTM1	2.6297355	up	osteopetrosis associated transmembrane protein 1
PPP1R2P4	2.6104445	up	protein phosphatase 1, regulatory (inhibitor) subunit 2 pseudogene 4
CTH	2.604754	up	cystathionase (cystathionine gamma-lyase)
GABARAPL1	2.603561	up	GABA(A) receptor-associated protein like 1
AHCY	2.6000764	up	S-adenosylhomocysteine hydrolase
CD209	2.5830662	up	CD209 molecule
GIMAP6	2.5744946	up	GTPase, IMAP family member 6
TFPI	2.566035	up	tissue factor pathway inhibitor (lipoprotein-associated coagulation inhibitor)
KCNS2	2.5646052	up	potassium voltage-gated channel, delayed-rectifier, subfamily S, member 2
SCCPDH	2.5630279	up	saccharopine dehydrogenase (putative)
CCDC69	2.5594425	up	coiled-coil domain containing 69
SYNGR1	2.5558324	up	synaptogyrin 1
CCDC90A	2.5552714	up	Coiled-coil domain containing 90A
EPAS1	2.5451322	up	endothelial PAS domain protein 1
LMO7	2.5414927	up	LIM domain 7
TCP11L1	2.5381732	up	t-complex 11 (mouse)-like 1
PLAGL1	2.533782	up	pleiomorphic adenoma gene-like 1
FYN	2.5213308	up	FYN oncogene related to SRC, FGR, YES
GPRC5B	2.5200324	up	G protein-coupled receptor, family C, group 5, member B
KLHL13	2.511186	up	kelch-like 13 (Drosophila)
FBN1	2.5067554	up	fibrillin 1
COX7A1	2.5002906	up	cytochrome c oxidase subunit VIIa polypeptide 1 (muscle)
SPIRE1	2.4959958	up	spire homolog 1 (Drosophila)
CYBRD1	2.4949534	up	cytochrome b reductase 1
CST7	2.4907544	up	cystatin F (leukocystatin)
KCNK4	2.4904733	up	potassium channel, subfamily K, member 4
PRKCH	2.487254	up	protein kinase C, eta
SRPX	2.486885	up	sushi-repeat-containing protein, X-linked
COPZ2	2.4824235	up	coatomer protein complex, subunit zeta 2
SEP11	2.4708505	up	septin 11
SPTLC3	2.4690392	up	serine palmitoyltransferase, long chain base subunit 3
FABP3	2.4685025	up	fatty acid binding protein 3, muscle and heart (mammary-derived growth inhibitor)
WARS	2.4607365	up	tryptophanyl-tRNA synthetase
ELMO1	2.4581966	up	engulfment and cell motility 1
HIST2H2AA3 /// HIST2H2AA4	2.456964	up	histone cluster 2, H2aa3 /// histone cluster 2, H2aa4
HIST1H2AC	2.4481454	up	histone cluster 1, H2ac
SNX29	2.4472013	up	sorting nexin 29
TTPAL	2.4392931	up	tocopherol (alpha) transfer protein-like
PSTK	2.4342778	up	phosphoseryl-tRNA kinase
PCBP3	2.4331248	up	poly(rC) binding protein 3
NMNAT2	2.4277217	up	nicotinamide nucleotide adenylyltransferase 2
COBLL1	2.421509	up	COBL-like 1
FICD	2.4130938	up	FIC domain containing
SNX25	2.4011533	up	sorting nexin 25
STS	2.3975098	up	steroid sulfatase (microsomal), isozyme S
GUCY1B3	2.39245	up	guanylate cyclase 1, soluble, beta 3
DYSF	2.3849163	up	dysferlin, limb girdle muscular dystrophy 2B (autosomal recessive)
RRAS	2.3811252	up	related RAS viral (r-ras) oncogene homolog
TNFRSF18	2.3808022	up	tumor necrosis factor receptor superfamily, member 18
RBKS	2.379315	up	ribokinase
RNF125	2.379253	up	ring finger protein 125
STOM	2.3749764	up	stomatin
IL1RL2	2.372242	up	interleukin 1 receptor-like 2
SRD5A1	2.3710213	up	steroid-5-alpha-reductase, alpha polypeptide 1 (3-oxo-5 alpha-steroid delta 4-dehydrogenase alpha 1)
NID1	2.3709407	up	nidogen 1
GOT1	2.3697505	up	glutamic-oxaloacetic transaminase 1, soluble (aspartate aminotransferase 1)
LAMA4	2.3618314	up	laminin, alpha 4
ADAMTS12	2.3614144	up	ADAM metallopeptidase with thrombospondin type 1 motif, 12
KCNB1	2.360776	up	potassium voltage-gated channel, Shab-related subfamily, member 1
FLRT3	2.3574924	up	fibronectin leucine rich transmembrane protein 3
GNPTAB	2.3469465	up	N-acetylglucosamine-1-phosphate transferase, alpha and beta subunits
ACOT8	2.3441532	up	Acyl-CoA thioesterase 8
PLCD1	2.3330145	up	phospholipase C, delta 1
KCTD21	2.3312242	up	potassium channel tetramerisation domain containing 21
MAGI3	2.3285232	up	membrane associated guanylate kinase, WW and PDZ domain containing 3
MAMLD1	2.3270473	up	mastermind-like domain containing 1
FHL5	2.3230145	up	four and a half LIM domains 5

OAS2	2.3213804	up	2'-5'-oligoadenylate synthetase 2, 69/71kDa
TRIOBP	2.318135	up	TRIO and F-actin binding protein
GLUD1	2.3136144	up	glutamate dehydrogenase 1
RORB	2.3111603	up	RAR-related orphan receptor B
COTL1	2.3062913	up	coactosin-like 1 (Dictyostelium)
SEC11C	2.305927	up	SEC11 homolog C (S. cerevisiae)
OSMR	2.2969682	up	oncostatin M receptor
RAB40B	2.2947042	up	RAB40B, member RAS oncogene family
P2RY1	2.2912707	up	purinergic receptor P2Y, G-protein coupled, 1
EMP3	2.2892659	up	epithelial membrane protein 3
SLC27A3	2.2821984	up	solute carrier family 27 (fatty acid transporter), member 3
HIST1H2BE	2.2715266	up	histone cluster 1, H2be
GATSL3	2.2702634	up	GATS protein-like 3
RIOK3	2.26686	up	RIO kinase 3 (yeast)
SBDS /// SBDSP	2.2610161	up	Shwachman-Bodian-Diamond syndrome /// Shwachman-Bodian-Diamond syndrome pseudogene
KRT86	2.2599013	up	keratin 86
PLCG2	2.2581644	up	phospholipase C, gamma 2 (phosphatidylinositol-specific)
FAM149A	2.2549262	up	family with sequence similarity 149, member A
SNAP29	2.2442958	up	synaptosomal-associated protein, 29kDa
SLC25A43	2.2436507	up	solute carrier family 25, member 43
FXYD5	2.2343013	up	FXYD domain containing ion transport regulator 5
CHST3	2.2326102	up	carbohydrate (chondroitin 6) sulfotransferase 3
CMC1	2.227872	up	COX assembly mitochondrial protein homolog (S. cerevisiae)
HIST1H4B	2.225741	up	Histone cluster 1, H4b
FAM167B	2.22102	up	family with sequence similarity 167, member B
RAB7L1	2.2192626	up	RAB7, member RAS oncogene family-like 1
CYSLTR1	2.2163198	up	cysteinyl leukotriene receptor 1
NOS3	2.2158537	up	nitric oxide synthase 3 (endothelial cell)
ACSL1	2.2142446	up	acyl-CoA synthetase long-chain family member 1
CTSA	2.2133102	up	cathepsin A
C1R	2.2115264	up	complement component 1, r subcomponent
CXCR3	2.2094607	up	chemokine (C-X-C motif) receptor 3
KIF16B	2.2087858	up	kinesin family member 16B
FKBP11	2.208326	up	FK506 binding protein 11, 19 kDa
LRMP	2.2054408	up	lymphoid-restricted membrane protein
OBFC1	2.2029107	up	oligonucleotide/oligosaccharide-binding fold containing 1
SH3BGRL3	2.1995866	up	SH3 domain binding glutamic acid-rich protein like 3
PCSK6	2.1953027	up	proprotein convertase subtilisin/kexin type 6
TCF7L2	2.1869316	up	transcription factor 7-like 2 (T-cell specific, HMG-box)
FGD5	2.1849988	up	FYVE, RhoGEF and PH domain containing 5
SH3D19	2.1758943	up	SH3 domain containing 19
PRSS23	2.1717389	up	Protease, serine, 23
ZBTB7C	2.1696367	up	zinc finger and BTB domain containing 7C
MAN1A2	2.166316	up	mannosidase, alpha, class 1A, member 2
MR1	2.1653214	up	major histocompatibility complex, class I-related
TSPAN5	2.1606061	up	tetraspanin 5
SPCS3	2.1568944	up	signal peptidase complex subunit 3 homolog (S. cerevisiae)
PPPDE2	2.1540978	up	PPPDE peptidase domain containing 2
METRN	2.1536465	up	meteorin, glial cell differentiation regulator
SLC44A2	2.1478071	up	solute carrier family 44, member 2
MRPL37	2.1449487	up	mitochondrial ribosomal protein L37
KIAA0513	2.1440537	up	KIAA0513
RORA	2.1427689	up	RAR-related orphan receptor A
ARL4D	2.135003	up	ADP-ribosylation factor-like 4D
ARAP2	2.1322567	up	ArfGAP with RhoGAP domain, ankyrin repeat and PH domain 2
ADCY3	2.1321876	up	adenylate cyclase 3
CPXM1	2.1234138	up	carboxypeptidase X (M14 family), member 1
TXNL4B	2.123364	up	thioredoxin-like 4B
MAPK1	2.1194506	up	mitogen-activated protein kinase 1
DTX4	2.118705	up	deltex homolog 4 (Drosophila)
KCNK6	2.1176696	up	potassium channel, subfamily K, member 6
HIST1H2BF	2.1138477	up	histone cluster 1, H2bf
KIAA0040	2.1109405	up	KIAA0040
PDLIM1	2.107823	up	PDZ and LIM domain 1
IVNS1ABP	2.105289	up	influenza virus NS1A binding protein
BRE	2.100276	up	brain and reproductive organ-expressed (TNFRSF1A modulator)
ERMP1	2.0886617	up	endoplasmic reticulum metallopeptidase 1
SETDB2	2.0839531	up	SET domain, bifurcated 2
PYGL	2.0839388	up	phosphorylase, glycogen, liver
SGK2	2.06848	up	serum/glucocorticoid regulated kinase 2
TSPAN4	2.0683057	up	tetraspanin 4
RAB33A	2.064811	up	RAB33A, member RAS oncogene family
BCORL1	2.061979	up	BCL6 co-repressor-like 1
SLC16A7	2.0524557	up	solute carrier family 16, member 7 (monocarboxylic acid transporter 2)
MAPK14	2.047455	up	mitogen-activated protein kinase 14
APOL6	2.0421255	up	apolipoprotein L, 6
ZNF846	2.0371647	up	zinc finger protein 846
PIGK	2.0338337	up	phosphatidylinositol glycan anchor biosynthesis, class K
HIST1H2BI	2.0312548	up	histone cluster 1, H2bi
MFGE8	2.0306673	up	milk fat globule-EGF factor 8 protein
APOC2 /// APOC4	2.0293102	up	apolipoprotein C-II /// apolipoprotein C-IV
STXBP1	2.0249867	up	syntaxin binding protein 1
GHR	2.0140123	up	growth hormone receptor
RFTN1	2.0132072	up	raftlin, lipid raft linker 1
PIK3R6	2.010077	up	phosphoinositide-3-kinase, regulatory subunit 6
WDR41	2.0098615	up	WD repeat domain 41
ALDH1L2	2.0048482	up	Aldehyde dehydrogenase 1 family, member L2
ENDOD1	2.0021384	up	endonuclease domain containing 1
RAMP3	2.0003152	up	receptor (G protein-coupled) activity modifying protein 3
