# Supplementary material for: Ectopic Pregnancy as a Model to Identify Endometrial Genes and Signaling Pathways Important in Decidualization and Regulated by Local Trophoblast
Source: PLoS One. 2011 Aug 17;6(8):e23595. doi: 10.1371/journal.pone.0023595 (PMC3157392; doi:10.1371/journal.pone.0023595)
Supplement: Table S2 — Top 20 canonical pathways associated with the array results comparing the decidua from women with ectopic pregnancies with little or no decidualization, and the decidua from women with ectopic pregnancies with moderate decidualization. (PDF) [file pone.0023595.s002.pdf]

Canonical Pathways - Decidualisation	P-value	Ratio
Natural Killer Cell Signaling	2.97 E-13	25/112
Wnt/beta-catenin Signaling	9.11 E-06	20/169
Crosstalk between Dendritic Cells and Natural Killer Cells	1.54 E-05	14/98
Melatonin Signaling	8.10 E-05	11/77
Role of Macrophages, Fibroblasts and Endothelial Cells in Rheumatoid Arthritis	1.14 E-04	28/357
Factors Promoting Cardiogenesis in Vertebrates	1.47 E-04	12/94
Glioblastoma Multiforme Signaling	2.15 E-04	16/163
Thyroid Cancer Signaling	3.52 E-04	8/47
Corticotropin Releasing Hormone Signaling	4.63 E-04	13/136
Thrombin Signaling	5.21 E-04	18/204
Cytotoxic T Lymphocyte-mediated Apoptosis of Target Cells	5.61 E-04	6/33
Human Embryonic Stem Cell Pluripotency	7.24 E-04	14/153
Molecular Mechanisms of Cancer	8.48 E-04	26/372
Role of Osteoblasts, Osteoclasts and Chondrocytes in Rheumatoid Arthritis	8.8 E-04	19/234
CCR5 Signaling in Macrophages	1.43 E-03	9/93
Basal Cell Carcinoma Signaling	1.76 E-03	9/73
Ovarian Cancer Signaling	2.03 E-03	13/140
PPAR-alpha/RXR-alpha Activation	2.19 E-03	15/180
Alpha-Adrenergic Signaling	2.56 E-03	10/106
Glycine, Serine and Threonine Metabolism	2.6 E-03	9/150
