# Supplementary material for: Ectopic Pregnancy as a Model to Identify Endometrial Genes and Signaling Pathways Important in Decidualization and Regulated by Local Trophoblast
Source: PLoS One. 2011 Aug 17;6(8):e23595. doi: 10.1371/journal.pone.0023595 (PMC3157392; doi:10.1371/journal.pone.0023595)
Supplement: Table S3 — List of all 85 genes with a FC≥2 derived from the comparison of the array results from decidua from women with ectopic pregnancies with moderate decidualization and decidua from women with intrauterine pregnancies with the same degree of decidualization. (PDF) [file pone.0023595.s003.pdf]

Gene Symbol	Fold change	Regulation	Gene Title
CSH1	7.626508	up	chorionic somatomammotropin hormone 1 (placental lactogen)
CSH2	7.4376197	up	chorionic somatomammotropin hormone 2
CPA3	5.658955	up	carboxypeptidase A3 (mast cell)
CGA	5.4369693	up	glycoprotein hormones, alpha polypeptide
CSHL1	4.9672112	up	chorionic somatomammotropin hormone-like 1
FOXQ1	4.9405756	up	forkhead box Q1
TPSAB1	4.860036	up	tryptase alpha/beta 1
TPSB2	4.552017	up	tryptase beta 2
POSTN	3.9408238	up	periostin, osteoblast specific factor
ZPLD1	3.9084785	up	zona pellucida-like domain containing 1
IFIT1	3.548241	up	interferon-induced protein with tetratricopeptide repeats 1
RASD1	3.3154707	up	RAS, dexamethasone-induced 1
AGTR1	3.16862	up	angiotensin II receptor, type 1
RSAD2	2.8885033	up	radical S-adenosyl methionine domain containing 2
CXCL10	2.8336082	up	chemokine (C-X-C motif) ligand 10
ITGB3	2.7670164	up	integrin, beta 3 (platelet glycoprotein IIIa, antigen CD61)
NTN4	2.7653468	up	netrin 4
CYP24A1	2.739624	up	cytochrome P450, family 24, subfamily A, polypeptide 1
GABBR1 /// UBD	2.7229636	up	gamma-aminobutyric acid (GABA) B receptor, 1 /// ubiquitin D
OBP2A /// OBP2B	2.6988323	up	odorant binding protein 2A /// odorant binding protein 2B
GPR110	2.6908078	up	G protein-coupled receptor 110
CXCL9	2.6107235	up	chemokine (C-X-C motif) ligand 9
CYP3A5	2.595368	up	cytochrome P450, family 3, subfamily A, polypeptide 5
HSD11B2	2.5401309	up	hydroxysteroid (11-beta) dehydrogenase 2
LAMP3	2.432947	up	lysosomal-associated membrane protein 3
CAMK2N1	2.407012	up	calcium/calmodulin-dependent protein kinase II inhibitor 1
SFTA2	2.3903527	up	surfactant associated 2
INHBB	2.349563	up	inhibin, beta B
OLIG3	2.3272738	up	oligodendrocyte transcription factor 3
TNFSF10	2.3216846	up	tumor necrosis factor (ligand) superfamily, member 10
PPP1R3C	2.2960248	up	protein phosphatase 1, regulatory (inhibitor) subunit 3C
CRTAM	2.2503574	up	cytotoxic and regulatory T cell molecule
CPA4	2.2324955	up	carboxypeptidase A4
LTBP1	2.2160273	up	latent transforming growth factor beta binding protein 1
DEFB1	2.2027926	up	defensin, beta 1
IL7R	2.1948836	up	interleukin 7 receptor
HES1	2.177035	up	hairy and enhancer of split 1, (Drosophila)
VPS41	2.1651795	up	vacuolar protein sorting 41 homolog (S. cerevisiae)
KHDRBS3	2.143701	up	KH domain containing, RNA binding, signal transduction associated 3
IGFBP3	2.1331255	up	insulin-like growth factor binding protein 3
CYS1	2.1305697	up	cystin 1
PROM1	2.1265528	up	prominin 1
TSPAN12	2.1248567	up	tetraspanin 12
XCL1 /// XCL2	2.1108098	up	chemokine (C motif) ligand 1 /// chemokine (C motif) ligand 2
LRRC32	2.1086445	up	leucine rich repeat containing 32
SLC25A35	2.0970516	up	solute carrier family 25, member 35
RHOB	2.0949848	up	ras homolog gene family, member B
MOBKL2B	2.077872	up	MOB1, Mps One Binder kinase activator-like 2B (yeast)
ARL4C	2.0738785	up	ADP-ribosylation factor-like 4C
CCL18	2.0736487	up	chemokine (C-C motif) ligand 18 (pulmonary and activation-regulated)
CITED2	2.055276	up	Cbp/p300-interacting transactivator, with Glu/Asp-rich carboxy-terminal domain, 2
HS6ST2	2.0365572	up	heparan sulfate 6-O-sulfotransferase 2
PER3	2.0331786	up	period homolog 3 (Drosophila)
FAM107A	2.0282586	up	family with sequence similarity 107, member A
HLA-DPA1	2.0255654	up	major histocompatibility complex, class II, DP alpha 1
FABP4	2.0102398	up	fatty acid binding protein 4, adipocyte
CRISP3	8.161555	down	cysteine-rich secretory protein 3
PSPH	6.0968385	down	phosphoserine phosphatase
UPK1B	4.4026856	down	uroplakin 1B
GAST	4.2205763	down	gastrin
GPC3	3.4669046	down	glypican 3
PTN	3.2119136	down	pleiotrophin
GABRA2	3.1759632	down	gamma-aminobutyric acid (GABA) A receptor, alpha 2
NOV	2.8469589	down	nephroblastoma overexpressed gene
NOG	2.8061116	down	noggin
LPA /// PLG	2.632613	down	lipoprotein, Lp(a) /// plasminogen
GRIA3	2.6318011	down	glutamate receptor, ionotrophic, AMPA 3
PSAT1	2.612459	down	phosphoserine aminotransferase 1
PLG	2.608607	down	plasminogen
UGT1A1 ///A10 ///A3 ///A4 ///A5 ///A6 ///A7 ///A8 ///A9	2.4913173	down	UDP glucuronosyltransferase 1 family, polypeptide A1 /// A10 /// A3 /// A4 /// A5 /// A6 /// A7 /// A8 /// A9
SLC7A11	2.485487	down	solute carrier family 7, (cationic amino acid transporter, y+ system) member 11
FLRT3	2.361156	down	fibronectin leucine rich transmembrane protein 3
HAP1	2.3476377	down	huntingtin-associated protein 1
LPHN3	2.29435	down	latrophilin 3
ATP7A	2.2459123	down	ATPase, Cu++ transporting, alpha polypeptide /// similar to ATPase, Cu++ transporting, alpha polypeptide
PEG10	2.2320137	down	paternally expressed 10
SNTN	2.2198198	down	sentan, cilia apical structure protein
DTX4	2.2097404	down	deltex homolog 4 (Drosophila)
ADAMTS12	2.1989598	down	ADAM metallopeptidase with thrombospondin type 1 motif, 12
OTUB2	2.1900342	down	OTU domain, ubiquitin aldehyde binding 2
PTPRD	2.0695236	down	protein tyrosine phosphatase, receptor type, D
MGST1	2.04794	down	Microsomal glutathione S-transferase 1
GBP3	2.0383294	down	guanylate binding protein 3
CXCL12	2.0367332	down	chemokine (C-X-C motif) ligand 12 (stromal cell-derived factor 1)
KCTD15	2.0117264	down	potassium channel tetramerisation domain containing 15
